# Supplementary material for: Association between high-fasting insulin levels and metabolic syndrome in non-diabetic middle-aged and elderly populations: a community-based study in Taiwan
Source: BMJ Open. 2018 May 3;8(5):e016554. doi: 10.1136/bmjopen-2017-016554 (PMC5942468; doi:10.1136/bmjopen-2017-016554)
Supplement: Author's manuscript [file bmjopen-2017-016554.draft_revisions.pdf]

BMJ Open is committed to open peer review. As part of this commitment we make the peer review history of every article we publish publicly available.

When an article is published we post the peer reviewers' comments and the authors' responses online. We also post the versions of the paper that were used during peer review. These are the versions that the peer review comments apply to.

The versions of the paper that follow are the versions that were submitted during the peer review process. They are not the versions of record or the final published versions. They should not be cited or distributed as the published version of this manuscript.

BMJ Open is an open access journal and the full, final, typeset and author-corrected version of record of the manuscript is available on our site with no access controls, subscription charges or pay-per-view fees (http://bmjopen.bmj.com).

If you have any questions on BMJ Open's open peer review process please email editorial.bmjopen@bmj.com

BMJ Open

High fasting insulin levels are associated with metabolic syndrome in non-diabetic middle-aged and elderly populations: A community-based study in Taiwan

Journal:	*BMJ Open*
Manuscript ID	bmjopen-2017-016554
Article Type:	Research
Date Submitted by the Author:	14-Mar-2017
Complete List of Authors:	Chen, Yun-Hung; Chang Gung Memorial Hospital Linkou Branch, Family Medicine<br>Lee, Yu-Chien; Chang Gung Memorial Hospital Linkou Branch, Family Medicine<br>Tsao, Yu-Chung; Chang Gung Memorial Hospital Linkou Branch, Family Medicine; Chang Gung Memorial Hospital Linkou Branch, Occupational Medicine<br>Lu, Mei-Chun; Chang Gung Memorial Hospital Linkou Branch, Family Medicine<br>Chuang, Hai-Hua; Chang Gung Memorial Hospital Taipei Branch, Family Medicine<br>Yeh, Wei-Chung; Chang Gung Memorial Hospital Linkou Branch, Family Medicine; Chang Gung University College of Medicine<br>Tzeng, I-Shiang; National Taipei University, Statistic<br>Chen, Jau-Yuan; Chang Gung Memorial Hospital Linkou Branch, Family Medicine; Chang Gung University College of Medicine
**Primary Subject Heading**:	General practice / Family practice
Secondary Subject Heading:	General practice / Family practice, Geriatric medicine, Health policy
Keywords:	metabolic syndrome, insulin resistance, fasting insulin level

SCHOLARONE™
Manuscripts

- **Title**

High fasting insulin levels are associated with metabolic syndrome in non-diabetic middle-aged and elderly populations: A community-based study in Taiwan

- **Short title**

Increased fasting insulin levels in association with metabolic syndrome

- **Author names and affiliations**

Yun-Hung Chen[1], Yu-Chien Lee[1], Yu-Chung Tsao[1, 2, 3], Mei-Chun Lu[1], Hai-Hua Chuang[4], Wei-Chung Yeh[1, 3], I-Shiang Tzeng[5], Jau-Yuan Chen[1, 3, *]

[1]Department of Family Medicine, Chang Gung Memorial Hospital, Linkou Branch, Taiwan

[2]Department of Occupational Medicine, Chang-Gung Memorial Hospital, Linkou Branch, Taiwan

[3]College of Medicine, Chang Gung University, Taoyuan, Taiwan

[4]Department of Family Medicine, Chang-Gung Memorial Hospital, Taipei Branch, Taiwan

[5]Department of Statistic, National Taipei University, Taiwan

*Corresponding author

E-mail: welins@cgmh.org.tw (J.-Y. Chen)

Postal address : No. 5, Fuxing St., Guishan Dist., Taoyuan City 333, Taiwan (R.O.C.)

Phone: +886-975362672

Fax: +886-3-3287715

- **Format**

Word count: 2252

Number of black and white figures: 2

Number of Tables: 5

Number of References: 31

For peer review only

ABSTRACT

Objectives: We determined the relevance between fasting insulin levels and metabolic syndrome (MetS), and suggested a value of fasting insulin (FI) which can help detect possible comorbidity of MetS in non-diabetic middle-aged and elder adults.

Design: Cross-sectional observational study.

Setting: Community-based investigation in Guishan township of northern Taiwan.

Participants: Our study included adults aged 50 years and above during community health exams between January and October 2014. People with diabetes mellitus were excluded. A total of 321 people were enrolled.

Outcome measures: We divided participants according to tertiles of FI as low, medium, and high levels. Pearson correlation was assessed between insulin level and each of the diagnostic components of metabolic syndrome (MetS-DCs) with adjustment of age. The prevalence of MetS-DCs based on tertiles of insulin levels were studied and analyzed by Cochran-Armitage trend test. The risk for prevalence of MetS in the middle, and high insulin group as compared to the low insulin group were assessed by multivariate logistic regression with adjustments for age, gender, smoking, body mass index, hypertension, and hyperlipidemia. Youden Index was performed for the optimized cut-off value.

Results: Our results showed positive correlation of FI level with systolic blood pressure, waist circumference, fasting plasma glucose and triglyceride levels, while negative correlation was shown with high-density lipoprotein ($p<0.001$). The prevalence of each MetS-DCs increased as a trend while FI levels increased ($p<0.001$). OR (95% CI) of MetS was 5.04 (2.15-11.81) for high insulin groups as compared to the low insulin group after adjusting multiple factors ($p<0.001$). AUC was 0.78, and cut-off value 7.35μU/mL for FI was obtained (sensitivity: 0.69; specificity: 0.77).

Conclusions: People with increased FI are associated with a higher prevalence of MetS. The

For peer review only - http://bmjopen.bmj.com/site/about/guidelines.xhtml

proposed cut-off value of FI may act as a marker for increased risk of MetS in middle-aged and elderly non-diabetic adults.

Keywords: metabolic syndrome, insulin resistance, fasting insulin level

Strengths and limitations of this study:

- This is the first study to explore the relationship between fasting insulin and MetS in the middle-aged and elderly populations of non-diabetic Asian people.
- We offer an easily measured biomarker to identify Chinese middle-aged and elderly with MetS.
- We adjusted many confounding factors to make the results more reliable.
- The recall bias from self-reported lifestyle behaviors are unavoidable.
- The causal relationship between fasting insulin level and MetS can't be demonstrated in our cross-sectional study.

For peer review only

INTRODUCTION

Metabolic syndrome (MetS) is associated with a cluster of unhealthy metabolic risk factors, including abdominal obesity (excess body fat around the waist), glucose intolerance, pre-morbid hypertension, and dyslipidemia [1-3]. A number of studies have reported that MetS increases the risk of type 2 diabetes mellitus (T2DM), cardiovascular disease (CVD) and other non-communicable diseases (NCDs) [4-7]. The rising prevalence of MetS has created a disturbing challenge to personal health [8-12].

Insulin resistance has long been associated with MetS [13-16]. Basal insulin represents 45%-50% of daily insulin [17,18]. The fasting insulin level approximates basal insulin [18,19], and the level increases as the body mass index (BMI) increases [20]. A number of studies have shown that elevated fasting insulin levels may predict the development of MetS [19,21]. We therefore determined the relationships between fasting plasma insulin and MetS in middle-aged and elderly populations. In addition, we also determined whether or not there is a cut-off value for fasting insulin as a predictive factor for the existence of MetS. By determining a cutoff value for fasting insulin, we could educate patients at risk for MetS and recommend aggressive lifestyle modification at an earlier stage.

METHODS

Study subjects

This was an observational and cross-sectional study conducted at Linkou Chang Gung Memorial Hospital in Taoyuan County, Taiwan between January and October 2014. Residents, 50-90 years of age, were eligible for the study. Participants diagnosed with diabetes mellitus were excluded. A total of 321 participants (111 males and 210 females) were ultimately enrolled for analysis. This study was approved by the Institutional Review Board of the hospital and written inform consent was obtained from all of the participants

For peer review only - http://bmjopen.bmj.com/site/about/guidelines.xhtml

before enrollment.

Data collection

We obtained exercise (exercising ≥3 times a week or not) and dietary habits (vegetarian or not) from self-administered questionnaires, which also included smoking (current smoker or not) and marital status (currently married or not). Anthropometric data, such as height, weight, waist circumference (WC), and blood pressure were also recorded. The subjects were dressed in light clothing without shoes for weight and height measurements. The BMI was calculated as the weight in kilograms (kg) divided by the height in meters squared (m^2). Waist circumference was measured midway between the inferior margin of the lowest rib and the iliac crest in the horizontal plane while in an upright position. Systolic blood pressure (SBP) and diastolic blood pressure (DBP) were checked at least 2 times after 5 min of rest while seated. Fasting insulin levels, lipid profile, and fasting glucose were obtained by blood sampling after a 10-h overnight fast. Blood samples were analyzed in the central laboratory of Linkou Chang Gung Memorial Hospital for fasting plasma glucose (FPG), serum total cholesterol (TC), low-density lipoprotein-cholesterol (LDL-C), high-density lipoprotein-cholesterol (HDL-C), serum triglycerides (TG), and fasting insulin levels.

Defining MetS

MetS was defined by at least 3 of 5 metabolic syndrome diagnostic components (MetS-DCs), according to The Third Report of the National Cholesterol Education Program Expert Panel (NCEP) on Adult Treatment Panel (ATP III) Asian diagnostic criteria [22]. The five MetS-DCs were as follows: 1) SBP ≥ 130 mmHg and/or DBP ≥ 85 mmHg, or the use of anti-hypertensive drugs; 2) decreased serum HDL-C concentration < 40 mg/dl in men and <50 mg/dL in women, or treatment for dyslipidemia; 3) TG concentration ≥ 150 mg/dl, or on medication for hypertriglyceridemia; 4) hyperglycemia: fasting plasma glucose level ≥ 100 mg/dl, previously diagnosed with diabetes mellitus, or on medication for diabetes mellitus;

For peer review only - http://bmjopen.bmj.com/site/about/guidelines.xhtml

For peer review only

and 5) abdominal waist circumference ≥ 90 cm in men or ≥ 80 cm in women.

Statistical Analysis

Subjects were classified in one of three groups according to serum insulin level tertiles as the low, middle, and high insulin groups. Clinical characteristics were expressed as the mean ± standard deviation (SD) for continuous variables and number (%) for categorical variables. One-way analysis of variance (ANOVA) or a Chi-square test was used to determine p-values for continuous or categorical variables, respectively. Pearson's correlation was performed for each MetS-DC in relation to fasting insulin levels. The Cochran-Armitage trend test was used to evaluate the increasing prevalence of MetS-DCs as a function of insulin level tertile. The low fasting insulin group was designated as the reference group to calculate the ORs of the prevalence of MetS in the middle and high fasting insulin groups. The trend test was used to identify the association between the fasting insulin level with the prevalence of MetS. A receiver operating characteristic curve (ROC) curve was created for fasting insulin as a predictor of MetS. The area under the ROC curve (AUC) was analyzed, and the optimized cut-off point for fasting insulin, sensitivity, and specificity were acquired using the maximal Youden's Index. We used SPSS (version 23.0 for windows), to perform the statistical analysis. Statistical significance was set at a p-value < 0.05.

RESULTS

A total of 321 individuals, 111 men (34.6%) and 210 (65.4%) women, with a mean age of 63.91±8.32 years, were enrolled in the current study. There were 90 study participants (28%) who met the diagnosis of MetS.

Table 1 shows the characteristics of the study population, which was divided based on the fasting insulin level in μU/mL. There were no statistically significant differences in age or gender between the low, middle, and high insulin level groups, while differences did exist with respect to WC, SBP, HDL-C, TG, and the proportion with MetS. Table 2 further shows

For peer review only - http://bmjopen.bmj.com/site/about/guidelines.xhtml

the correlation between the fasting insulin level and all MetS-DCs, even after adjusting for age. Fasting insulin was positively correlated with SBP, WC, FPG, and TG, and negatively correlated with HDL-C, as shown in Table 2. Table 3 shows the prevalence of MetS-DCs (hypertension, hyperglycemia, dyslipidemia, and central obesity) according to the insulin level tertiles. The prevalence of MetS-DCs increased as the fasting insulin level increased, as shown by significant p values (Cochran-Armitage trend test). Figure 1 shows that the low insulin level group had a 10% prevalence of MetS, the middle insulin level group had a 21.5% prevalence of MetS, and the high insulin level group had a 53.8% of MetS ($p<0.0001$ [Cochran-Armitage trend test]), suggesting that the prevalence of MetS increased with an increase in fasting insulin levels.

For peer review only

For peer review only - http://bmjopen.bmj.com/site/about/guidelines.xhtml

Table 1 General characteristics of the study population based on insulin levels.

Variables	Insulin levels				p value
	Total (n=321)	Low (n=110) (≦4.8)	Middle (n=107) (4.9-7.8)	High (n=104) (≧7.9)	
Age (year)	63.91 ±8.32	64.23 ±8.32	64.47 ±8.67	63.01 ±7.93	0.40
BMI (kg/m^2)	24.36 ±3.53	22.41 ±3.14	24.41 ±2.73	26.37 ±3.54	<0.001
Waist circumference (cm)	84.23 ±9.51	79.69 ±7.57	83.78 ±8.63	89.51 ±9.65	<0.001
SBP (mmHg)	129.02 ±16.59	123.69 ±17.36	129.52 ±14.51	134.13 ±16.20	<0.001
DBP (mmHg)	77.01 ±10.90	75.43 ±11.80	76.92 ±10.03	78.79 ±10.60	0.08
ALT (U/L)	21.74 ±11.06	18.94 ±7.81	20.33 ±9.25	26.15 ±14.05	<0.001
Creatinine (mg/dL)	0.76 ±0.44	0.69 ±0.17	0.85 ±0.66	0.75 ±0.34	0.03
FPG (mg/dL)	89.10 ±9.93	85.29 ±9.11	89.18 ±8.52	93.05 ±10.60	<0.001
HDL-C (mg/dL)	55.70 ±14.05	60.93 ±14.85	55.59 ±13.17	50.28 ±11.94	<0.001
Insulin (μU/mL)	7.10 ±4.14	3.60 ±0.94	6.21 ±0.86	11.72 ±4.02	<0.001
LDL-C (mg/dL)	121.48 ±32.05	118.90 ±34.55	126.03 ±31.01	119.53 ±30.10	0.20
T-cholesterol (mg/dL)	200.61 ±35.20	198.85 ±36.98	203.81 ±35.12	119.18 ±33.43	0.52
TG (mg/dL)	117.34 ±60.61	95.39 ±45.13	111.04 ±49.83	147.05 ±72.48	<0.001
Current smoking, n(%)	34 (10.6)	14 (12.7)	11 (10.3)	9 (8.7)	0.62
Marital status (single), n(%)	54 (16.8)	22 (20.0)	14 (13.1)	18 (17.3)	0.39
Men, n(%)	111 (34.6)	41 (37.3)	39 (36.4)	31 (29.8)	0.46
Regular exercise, n(%)	264 (82.2)	92 (83.6)	96 (89.7)	76 (73.1)	0.01
Vegetarian, n(%)	20 (6.2)	7 (6.4)	7 (6.5)	6 (5.8)	0.97
HTN, n(%)	150 (46.7)	43 (39.1)	47 (43.9)	60 (57.7)	0.02
Hyperlipidemia, n(%)	204 (63.6)	58 (52.7)	69 (64.5)	77 (74.0)	0.005
Metabolic syndrome, n(%)	90 (28.0)	11 (10.0)	23 (21.5)	56 (53.8)	<0.001

Notes: Clinical characteristics are expressed as the mean±SD for continuous variables and n(%) for categorical variables. P-values were derived from one-way analysis of variance (ANOVA) for continuous variables and chi-square test for categorical variables.

Abbreviations: BMI, body mass index; SBP, systolic blood pressure; DBP, diastolic blood

For peer review only - http://bmjopen.bmj.com/site/about/guidelines.xhtml

pressure; ALT, alanine aminotransferase; FPG, fasting plasma glucose; HDL-C, high-density lipoprotein cholesterol; LDL-C, low-density lipoprotein cholesterol; TG, triglyceride; HTN, hypertension.

When designating the low insulin level group as the reference, the middle and high insulin level groups had an OR of 2.46 (p=0.02) and 10.50 (p<0.001) for predicting MetS, respectively. After adjusting age, gender, and BMI, the middle and high insulin level groups had an OR of 1.71 (p= 0.20) and 5.63 (p <0.001) for predicting MetS, respectively. After adjusting age, gender, BMI, smoking, hypertension and dyslipidemia, the middle and high insulin level groups still had an OR of 1.51 (p=0.35) and 5.04 (p<0.001; Table 4) for predicting MetS, respectively. There was no significant difference between the middle and low tertile groups, but a significant difference between the high and low tertile groups, even after adjusting for the above risk factors. Based on this data, the high insulin level group had a 5-fold times risk for MetS compared to the low insulin level group.

Figure 2 shows the ROC curve of the insulin level as a predictor for MetS. The AUC was 0.78. The optimized cut-off value for insulin was shown to be 7.35 μU/mL, with a sensitivity of 0.69 and a specificity of 0.77 (Table 5).

For peer review only - http://bmjopen.bmj.com/site/about/guidelines.xhtml

DISCUSSION

In this community-based study, we investigated fasting serum insulin levels in association with the prevalence of MetS in non-diabetic middle-aged and elderly Taiwanese adults. In our study, the prevalence of MetS in the relatively healthy middle age-to-elderly population was 28%, which is similar to the 29.75% findings reported by Li et al.[23] among middle-aged and elderly populations in Taiwan. Looking at the three groups as a function of fasting insulin tertiles, there was a rising proportion of MetS as the fasting insulin level increased. This finding not only applied to MetS, but to MetS-DCs as well. The WC, SBP, TG, and FPG levels were the lowest in the low fasting insulin group and highest in the high fasting insulin group; the converse applied to HDL and vice versa (Table 1). This finding led us speculate that an association exists between fasting insulin levels and MetS-DCs. We found a statistically significant correlation between fasting insulin levels and each MetS-DC, even after adjusting for age (Table 2). A trend existed between the fasting insulin level and the prevalence of all five MetS-DCs (Table 3). We thus wanted to know if the trend applied to the prevalence of MetS. Figure 1 shows an apparent increase in the prevalence of MetS as the fasting insulin level increased. The trend was confirmed by the Cochran-Armitage trend test ($p<0.0001$).

For peer review only

Table 2 Pearson's correlation coefficients for each component of metabolic syndrome and age in relation to insulin levels.

Variables	Insulin(n=321)			
	Unadjusted		Adjusted for age	
	Pearson's coefficient	p value	Pearson's coefficient	p value
Age (year)	-0.04	0.50	NA	NA
SBP (mmHg)	0.21	<0.001	0.22	<0.001
DBP (mmHg)	0.11	0.05	0.10	0.07
Waist circumference (cm)	0.43	<0.001	0.44	<0.001
FPG (mg/dL)	0.38	<0.001	0.39	<0.001
HDL-C (mg/dL)	-0.37	<0.001	-0.37	<0.001
TG (mg/dL)	0.37	<0.001	0.37	<0.001

Abbreviations: SBP, systolic blood pressure; DBP, diastolic blood pressure; FPG, fasting plasma glucose; HDL-C, high-density lipoprotein cholesterol; TG, triglyceride.

For peer review only

Table 3 Prevalence of components of metabolic syndrome based on insulin levels.

**Components**	**Low (n=110) N(%)**	**Middle (n=107) N(%)**	**High (n=104) N(%)**	**p value for Cochran-Armitage trend test**
High blood pressure[a]	56(50.9)	63(58.9)	78(75)	0.0003
High blood glucose[b]	8(7.3)	10(9.3)	25(24.0)	0.0004
Low HDL-C[c]	15(13.6)	19(17.8)	43(41.3)	<0.0001
High TG[d]	17(15.5)	27(25.2)	42(40.4)	<0.0001
Central obesity[e]	34(30.9)	57(53.3)	82(78.8)	<0.0001

Note:

[a] SBP≧130 mmHg or DBP≧85 mmHg, or self-reported hypertension

[b] Fasting blood glucose ≥100 mg/dL or self-reported diabetes mellitus

[c] HDL-C<40 mg/dL in men or <50 mg/dL in women

[d] TG≥150 mg/dL

[e] Waist circumference ≥90 cm in men or ≥80 cm in women

Abbreviations: HDL-C, high-density lipoprotein-cholesterol; TG, triglyceride; SBP, systolic blood pressure; DBP, diastolic blood pressure.

For peer review only

For peer review only - http://bmjopen.bmj.com/site/about/guidelines.xhtml

After adjusting for gender, age, BMI, smoking status, hypertension, and dyslipidemia, the middle-aged and elderly populations in the high fasting insulin group were at significant risk for developing MetS (OR = 5.04, 95% CI =2.15-11.81; P < 0.01: Table 4). This conclusion is consistent with the finding of Sung et al. [19], who reported that higher fasting insulin levels increased the risk of acquiring MetS. One possible explanation might be the relationship between the fasting insulin level and insulin resistance, which has a fundamental role in MetS [24 25]. Although the mechanism by which fasting insulin may induce insulin resistance was not investigated in the present study, a number of studies have shown that fasting insulin is a suitable surrogate marker for insulin resistance [26-29], calculated by the fasting insulin resistance index (FIRI) or homeostasis model assessment of insulin resistance (HOMA-IR). Based on these equations, a higher fasting insulin level can contribute to insulin resistance in patients with impaired fasting glucose, but may be an inappropriate marker in diabetics with poor glycemic control. This finding may be the reason that a higher fasting insulin level increases the risk of acquiring MetS in non-diabetic populations. It has been reported that the fasting insulin level is highly associated with MetS, but a cutoff value was not established [21]. Based on our search of the literature, there is no widely accepted reference range for fasting insulin. A reference range for fasting insulin of 1.57–16.32 μU/mL has been proposed in Chinese men, but the reference range varies between different ethnicities and genders [30]. A fasting insulin level > 9μU/mL has been reported to identify 80% of patients with pre-diabetes [31]. In our study, the AUC for fasting insulin as a predictor for MetS was 0.78, and our proposed cut-off value for fasting insulin was 7.35 μU/mL (sensitivity=0.69 and specificity=0.77: Table 5). Because MetS is a state of pre-diabetes [25], our cutoff value < 9μU/mL is reasonable. Our proposed cut-off value of 7.35 μU/mL is between the mean fasting insulin levels of the middle and high tertile groups. This may be reasonable because the middle and low tertile groups did not show a statistically significant difference in OR for

For peer review only - http://bmjopen.bmj.com/site/about/guidelines.xhtml

MetS after adjusting for confounding factors.

For peer review only

For peer review only - http://bmjopen.bmj.com/site/about/guidelines.xhtml

Table 4 Association between insulin levels and metabolic syndrome.

Variables	Model 1			Model 2			Model 3		
	OR	(95%CI)	p value	OR	(95%CI)	p value	OR	(95%CI)	p value
Low	1.00	—	—	1.00	—	—	1.00	—	—
Middle	2.46	(1.14-5.35)	0.02	1.71	(0.76-3.85)	0.20	1.51	(0.64-3.57)	0.35
High	10.50	(5.05-21.84)	<0.001	5.63	(2.53-12.53)	<0.001	5.04	(2.15-11.81)	<0.001
p value for trend			<0.001			<0.001			<0.001

Model 1: unadjusted

Model 2: adjusted for gender, age, and BMI

Model 3: adjusted for factors in model 2 plus smoking, HTN, and hyperlipidemia

Abbreviations: BMI, body mass index; HTN, hypertension; OR, odds ratio; CI, confidence interval.

Table 5 The areas under the ROC curve (AUC), sensitivity, and specificity at the optimized cut-off point for insulin in predicting metabolic syndrome.

Variables	AUC(95% CI)	p value	Cut-off point	Sensitivity	Specificity
Insulin (μU/mL)	0.78(0.72-0.84)	<0.001	7.35	0.69	0.77

Abbreviations: ROC curve, receiver operating characteristic curve; CI, confidence interval.

For peer review only

Our findings may have an impact on health screening policies in people older than middle-age. Aggressive lifestyle modification should be promptly instituted when the fasting insulin level is > 7.35 μU/mL, and in the case of progression, some medical interventions should be considered.

Some limitations in our study merit consideration. First, the principal limitation relevant to the interpretation of our results was the use of a cross-sectional design, thus a causal relationship between the fasting insulin level and MetS cannot be inferred. Second, the sample size in our study was relatively small (n=321[the power was not calculated]) and the participants were recruited from a regional community. The participants could therefore only be distributed into three groups and the results cannot be generalized to other ethnicities. Furthermore, even though we used a standardized questionnaire, recall and reporting bias are unavoidable for self-reported data. Finally, we did not ask participants to sleep adequately or to avoid vigorous exercise the day before blood testing, which could affect the accuracy of the fasting serum insulin level.

Our study also has strengths. First, our participants were recruited during a community health examination and represent a relatively healthy population. The effects of important confounders, including ethnicity, residential area, and environmental factors, were minimized. Second, we used standardized laboratory examination protocols and anthropometric measurements. Third, while evaluating the association between MetS and the fasting insulin level, we excluded diabetic patients to avoid the effect of anti-diabetic medications on the fasting insulin level.

In the future, we will continue to follow this community and record the development of newly diagnosed MetS.

For peer review only

For peer review only - http://bmjopen.bmj.com/site/about/guidelines.xhtml

CONCLUSIONS

This study provides another convenient method to predict the existence of subsequent MetS by testing fasting insulin levels in the in the middle-aged and elderly non-diabetic populations. We suggest a cut-off value of 7.35 μU/mL for MetS. We believe that the cut-off value is a robust and reliable predictor which facilitates the early detection of MetS. Providing medical counseling to patients with a fasting insulin level > 7.35 μU/mL should result in long-term health benefits.

Contributors YHC and YCL were involved in writing of the manuscript. YCT, MCL, HHC and WCY conceived and supervised the study. IST provided statistical advice. JYC contributed conceived, designed and performed the experiments, collected and analyzed the data, revising it critically for important intellectual content and final approval of the version to be submitted.

Funding This work was supported by Chang Gung Memorial Hospital (CORPG3C0171, CORPG3C0172, CZRPG3C0053).

Competing interests None declared.

Ethics approval The study was approved by Chang-Gung Medical Foundation Institutional Review Board (102-2304B), and written informed consent was given by all the participants before enrollment.

Data sharing statement No additional data are available.

For peer review only

REFERENCES

1. Kaur J. A comprehensive review on metabolic syndrome. *Cardiol Res Pract* 2014;2014:943162. doi: 10.1155/2014/943162

2. Nakao YM, Miyawaki T, Yasuno S, et al. Intra-abdominal fat area is a predictor for new onset of individual components of metabolic syndrome: MEtabolic syndRome and abdominaL ObesiTy (MERLOT study). *Proc Jpn Acad Ser B Phys Biol Sci* 2012;88(8):454-61.

3. Popa S, Mota M, Popa A, et al. Prevalence of overweight/obesity, abdominal obesity and metabolic syndrome and atypical cardiometabolic phenotypes in the adult Romanian population: PREDATORR study. *J Endocrinol Invest* 2016;39(9):1045-53. doi: 10.1007/s40618-016-0470-4

4. Metabolic syndrome and mental illness. Weight gain and other unhealthy attributes increase risk of diabetes and heart disease. *Harv Ment Health Lett* 2011;28(2):5.

5. Dragsbaek K, Neergaard JS, Laursen JM, et al. Metabolic syndrome and subsequent risk of type 2 diabetes and cardiovascular disease in elderly women: Challenging the current definition. *Medicine (Baltimore)* 2016;95(36):e4806. doi: 10.1097/MD.0000000000004806

6. Meifang Y, Xue S, Jue H, et al. [Metabolic syndrome increases Framingham risk score of patients with type 2 diabetes mellitus]. *Zhejiang Da Xue Xue Bao Yi Xue Ban* 2016;45(3):268-74.

7. Oguoma VM, Nwose EU, Skinner TC, et al. Association between metabolic syndrome and 10-year risk of developing cardiovascular disease in a Nigerian population. *Int Health* 2016 doi: 10.1093/inthealth/ihw013

8. Desroches S, Lamarche B. The evolving definitions and increasing prevalence of the metabolic syndrome. *Appl Physiol Nutr Metab* 2007;32(1):23-32. doi: 10.1139/h06-095

For peer review only

For peer review only - http://bmjopen.bmj.com/site/about/guidelines.xhtml

9. Ford ES, Giles WH, Mokdad AH. Increasing prevalence of the metabolic syndrome among u.s. Adults. *Diabetes Care* 2004;27(10):2444-9.

10. Hu G, Lindstrom J, Jousilahti P, et al. The increasing prevalence of metabolic syndrome among Finnish men and women over a decade. *J Clin Endocrinol Metab* 2008;93(3):832-6. doi: 10.1210/jc.2007-1883

11. Lim S, Shin H, Song JH, et al. Increasing prevalence of metabolic syndrome in Korea: the Korean National Health and Nutrition Examination Survey for 1998-2007. *Diabetes Care* 2011;34(6):1323-8. doi: 10.2337/dc10-2109

12. Liu M, Wang J, Jiang B, et al. Increasing Prevalence of Metabolic Syndrome in a Chinese Elderly Population: 2001-2010. *PLoS One* 2013;8(6):e66233. doi: 10.1371/journal.pone.0066233

13. Grundy SM, Brewer HB, Jr., Cleeman JI, et al. Definition of metabolic syndrome: Report of the National Heart, Lung, and Blood Institute/American Heart Association conference on scientific issues related to definition. *Circulation* 2004;109(3):433-8. doi: 10.1161/01.CIR.0000111245.75752.C6

14. Lann D, LeRoith D. Insulin resistance as the underlying cause for the metabolic syndrome. *Med Clin North Am* 2007;91(6):1063-77, viii. doi: 10.1016/j.mcna.2007.06.012

15. Gallagher EJ, Leroith D, Karnieli E. Insulin resistance in obesity as the underlying cause for the metabolic syndrome. *Mt Sinai J Med* 2010;77(5):511-23. doi: 10.1002/msj.20212

16. Dontsov AV, Vasil'eva LV. [Insulin Resistance Associated with Metabolic Syndrome as an Indicator of Cardiovascular Risk]. *Klin Med (Mosk)* 2016;94(3):189-93.

17. Polonsky KS, Given BD, Van Cauter E. Twenty-four-hour profiles and pulsatile patterns of insulin secretion in normal and obese subjects. *J Clin Invest* 1988;81(2):442-8. doi: 10.1172/JCI113339

18. Wilcox G. Insulin and insulin resistance. *Clin Biochem Rev* 2005;26(2):19-39.

For peer review only - http://bmjopen.bmj.com/site/about/guidelines.xhtml

19. Sung KC, Seo MH, Rhee EJ, et al. Elevated fasting insulin predicts the future incidence of metabolic syndrome: a 5-year follow-up study. *Cardiovasc Diabetol* 2011;10:108. doi: 10.1186/1475-2840-10-108

20. Freedman DS, Kahn HS, Mei Z, et al. Relation of body mass index and waist-to-height ratio to cardiovascular disease risk factors in children and adolescents: the Bogalusa Heart Study. *Am J Clin Nutr* 2007;86(1):33-40.

21. Saravia G, Civeira F, Hurtado-Roca Y, et al. Glycated Hemoglobin, Fasting Insulin and the Metabolic Syndrome in Males. Cross-Sectional Analyses of the Aragon Workers' Health Study Baseline. *PLoS One* 2015;10(8):e0132244. doi: 10.1371/journal.pone.0132244

22. Tan CE, Ma S, Wai D, et al. Can we apply the National Cholesterol Education Program Adult Treatment Panel definition of the metabolic syndrome to Asians? *Diabetes Care* 2004;27(5):1182-6.

23. Li Z, Yang X, Yang J, et al. The Cohort Study on Prediction of Incidence of All-Cause Mortality by Metabolic Syndrome. *PLoS One* 2016;11(5):e0154990. doi: 10.1371/journal.pone.0154990

24. Saltiel AR. Series introduction: the molecular and physiological basis of insulin resistance: emerging implications for metabolic and cardiovascular diseases. *J Clin Invest* 2000;106(2):163-4. doi: 10.1172/JCI10533

25. Mayans L. Metabolic Syndrome: Insulin Resistance and Prediabetes. *FP Essent* 2015;435:11-6.

26. Duncan MH, Singh BM, Wise PH, et al. A simple measure of insulin resistance. *Lancet* 1995;346(8967):120-1.

27. Abbasi F, Reaven GM. Evaluation of the quantitative insulin sensitivity check index as an estimate of insulin sensitivity in humans. *Metabolism* 2002;51(2):235-7.

For peer review only

For peer review only - http://bmjopen.bmj.com/site/about/guidelines.xhtml

28. Kim SH, Abbasi F, Reaven GM. Impact of degree of obesity on surrogate estimates of insulin resistance. *Diabetes Care* 2004;27(8):1998-2002.

29. Tang Q, Li X, Song P, et al. Optimal cut-off values for the homeostasis model assessment of insulin resistance (HOMA-IR) and pre-diabetes screening: Developments in research and prospects for the future. *Drug Discov Ther* 2015;9(6):380-5. doi: 10.5582/ddt.2015.01207

30. Li S, Huang S, Mo ZN, et al. Generating a reference interval for fasting serum insulin in healthy nondiabetic adult Chinese men. *Singapore Med J* 2012;53(12):821-5.

31. Johnson JL, Duick DS, Chui MA, et al. Identifying prediabetes using fasting insulin levels. *Endocr Pract* 2010;16(1):47-52. doi: 10.4158/EP09031.OR

For peer review only

For peer review only - http://bmjopen.bmj.com/site/about/guidelines.xhtml

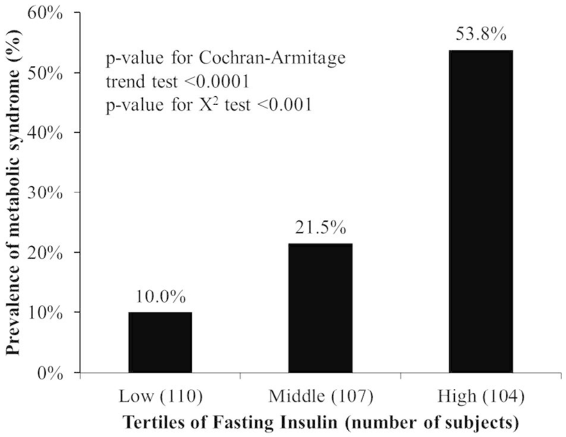

Figure 1 Prevalence of metabolic syndrome based on insulin levels. A linear increasing trend across insulin tertiles.

Figure 1 Prevalence of metabolic syndrome based on insulin levels. A linear increasing trend across insulin tertiles.

284x140mm (150 x 150 DPI)

For peer review only

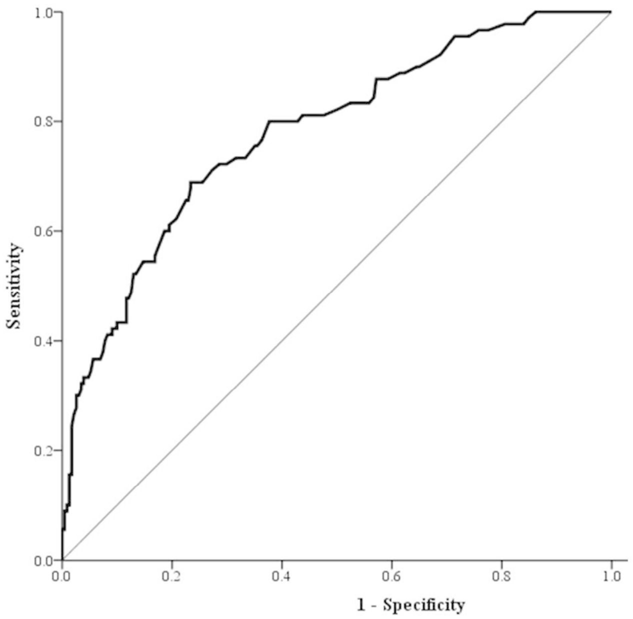

Figure 2 ROC curve for insulin as a predictor of metabolic syndrome.

Figure 2 ROC curve for insulin as a predictor of metabolic syndrome.

254x171mm (150 x 150 DPI)

STROBE 2007 (v4) Statement—Checklist of items that should be included in reports of *cross-sectional studies*

Section/Topic	Item #	Recommendation	Reported on page #
**Title and abstract**	1	(*a*) Indicate the study's design with a commonly used term in the title or the abstract	P2
		(*b*) Provide in the abstract an informative and balanced summary of what was done and what was found	P2
**Introduction**			
Background/rationale	2	Explain the scientific background and rationale for the investigation being reported	P4
Objectives	3	State specific objectives, including any prespecified hypotheses	P2, P4
**Methods**			
Study design	4	Present key elements of study design early in the paper	P4
Setting	5	Describe the setting, locations, and relevant dates, including periods of recruitment, exposure, follow-up, and data collection	P4, 5
Participants	6	(*a*) Give the eligibility criteria, and the sources and methods of selection of participants	P4
Variables	7	Clearly define all outcomes, exposures, predictors, potential confounders, and effect modifiers. Give diagnostic criteria, if applicable	P5
Data sources/ measurement	8*	For each variable of interest, give sources of data and details of methods of assessment (measurement). Describe comparability of assessment methods if there is more than one group	P5
Bias	9	Describe any efforts to address potential sources of bias	Nil
Study size	10	Explain how the study size was arrived at	Nil
Quantitative variables	11	Explain how quantitative variables were handled in the analyses. If applicable, describe which groupings were chosen and why	
Statistical methods	12	(*a*) Describe all statistical methods, including those used to control for confounding	P6
		(*b*) Describe any methods used to examine subgroups and interactions	P6
		(*c*) Explain how missing data were addressed	Nil
		(*d*) If applicable, describe analytical methods taking account of sampling strategy	Nil
		(*e*) Describe any sensitivity analyses	Nil
**Results**			

For peer review only

Participants	13*	(a) Report numbers of individuals at each stage of study—eg numbers potentially eligible, examined for eligibility, confirmed eligible, included in the study, completing follow-up, and analysed	Nil
		(b) Give reasons for non-participation at each stage	Nil
		(c) Consider use of a flow diagram	Nil
Descriptive data	14*	(a) Give characteristics of study participants (eg demographic, clinical, social) and information on exposures and potential confounders	P8
		(b) Indicate number of participants with missing data for each variable of interest	Nil
Outcome data	15*	Report numbers of outcome events or summary measures	P6-9
Main results	16	(*a*) Give unadjusted estimates and, if applicable, confounder-adjusted estimates and their precision (eg, 95% confidence interval). Make clear which confounders were adjusted for and why they were included	P7
		(*b*) Report category boundaries when continuous variables were categorized	P8
		(*c*) If relevant, consider translating estimates of relative risk into absolute risk for a meaningful time period	Nil
Other analyses	17	Report other analyses done—eg analyses of subgroups and interactions, and sensitivity analyses	Nil
**Discussion**			
Key results	18	Summarise key results with reference to study objectives	P10
Limitations	19	Discuss limitations of the study, taking into account sources of potential bias or imprecision. Discuss both direction and magnitude of any potential bias	P16
Interpretation	20	Give a cautious overall interpretation of results considering objectives, limitations, multiplicity of analyses, results from similar studies, and other relevant evidence	P13
Generalisability	21	Discuss the generalisability (external validity) of the study results	P16
**Other information**			
Funding	22	Give the source of funding and the role of the funders for the present study and, if applicable, for the original study on which the present article is based	P17

*Give information separately for cases and controls in case-control studies and, if applicable, for exposed and unexposed groups in cohort and cross-sectional studies.

Note: An Explanation and Elaboration article discusses each checklist item and gives methodological background and published examples of transparent reporting. The STROBE checklist is best used in conjunction with this article (freely available on the Web sites of PLoS Medicine at http://www.plosmedicine.org/, Annals of Internal Medicine at http://www.annals.org/, and Epidemiology at http://www.epidem.com/). Information on the STROBE Initiative is available at www.strobe-statement.org.

For peer review only

High fasting insulin levels are associated with metabolic syndrome in non-diabetic middle-aged and elderly populations: A community-based study in Taiwan

Journal:	*BMJ Open*
Manuscript ID	bmjopen-2017-016554.R1
Article Type:	Research
Date Submitted by the Author:	07-Sep-2017
Complete List of Authors:	Chen, Yun-Hung; Chang Gung Memorial Hospital Linkou Branch, Family Medicine<br>Lee, Yu-Chien; Chang Gung Memorial Hospital Linkou Branch, Family Medicine<br>Tsao, Yu-Chung; Chang Gung Memorial Hospital Linkou Branch, Family Medicine; Chang Gung Memorial Hospital Linkou Branch, Occupational Medicine<br>Lu, Mei-Chun; Chang Gung Memorial Hospital Linkou Branch, Family Medicine<br>Chuang, Hai-Hua; Chang Gung Memorial Hospital Taipei Branch, Family Medicine<br>Yeh, Wei-Chung; Chang Gung Memorial Hospital Linkou Branch, Family Medicine; Chang Gung University College of Medicine<br>Tzeng, I-Shiang; Taipei Tzu Chi General Hospital, Department of Research<br>Chen, Jau-Yuan; Chang Gung Memorial Hospital Linkou Branch, Family Medicine; Chang Gung University College of Medicine
**Primary Subject Heading**:	General practice / Family practice
Secondary Subject Heading:	General practice / Family practice, Geriatric medicine, Health policy
Keywords:	metabolic syndrome, insulin resistance, fasting insulin level

SCHOLARONE™
Manuscripts

- **Title**

High fasting insulin levels are associated with metabolic syndrome in non-diabetic middle-aged and elderly populations: A community-based study in Taiwan

- **Short title**

Increased fasting insulin levels in association with metabolic syndrome

- **Author names and affiliations**

Yun-Hung Chen[1], Yu-Chien Lee[1], Yu-Chung Tsao[1, 2, 3], Mei-Chun Lu[1], Hai-Hua Chuang[4], Wei-ChungYeh[1, 3], I-Shiang Tzeng[5], Jau-Yuan Chen[1, 3, *]

[1]Department of Family Medicine, Chang Gung Memorial Hospital, Linkou Branch, Taiwan

[2]Department of Occupational Medicine, Chang-Gung Memorial Hospital, Linkou Branch, Taiwan

[3]College of Medicine, Chang Gung University, Taoyuan, Taiwan

[4]Department of Family Medicine, Chang-Gung Memorial Hospital, Taipei Branch, Taiwan

[5]Department of Research, Taipei Tzu Chi Hospital, Buddhist Tzu Chi Medical Foundation, New Taipei city, Taiwan

*Corresponding author

E-mail: welins@cgmh.org.tw (J.-Y. Chen)

Postal address: No. 5, Fuxing St., Guishan Dist., Taoyuan City 333, Taiwan (R.O.C.)

Phone: +886-975362672

Fax: +886-3-3287715

- **Format**

Word count: 2659

Number of black and white figures: 2

Number of Tables: 5

Number of References: 41

For peer review only

ABSTRACT

Objectives: We determined the association on between fasting insulin (FI) levels and metabolic syndrome (MetS), and suggested a value of (FI) which can help detect possible comorbidity of MetS in non-diabetic middle-aged and elder adults.

Design: Cross-sectional observational study.

Setting: Community-based investigation in Guishantownship of northern Taiwan.

Participants: Our study included adults aged 50 years and above during community health exams between January and October 2014. People with diabetes mellitus were excluded. A total of 321 people were enrolled.

Outcome measures: We divided participants according to tertiles of FI as low, medium, and high levels. Pearson correlation was assessed between insulin level and each of the diagnostic components of metabolic syndrome (MetS-DCs) with adjustment of age. The prevalence of MetS-DCs based on tertiles of FI were studied and analyzed by Cochran-Armitage trend test. The risk for prevalence of MetS in the middle, and high insulin group as compared to the low insulin group were assessed by multivariate logistic regression with adjustments for age, gender, smoking, body mass index, hypertension, and hyperlipidemia. Youden Index was performed for the optimized cut-off value.

Results: Our results showed positive correlation of FI level with systolic blood pressure, waist circumference, fasting plasma glucose and triglyceride levels, while negative correlation was shown with high-density lipoprotein ($p<0.001$). The prevalence of each MetS-DCs increased as a trend while FI levels increased ($p<0.001$). OR (95% CI) of MetS was 5.04(2.15-11.81) for high insulin groups ~~as~~ compared to the low insulin group after adjusting confounders ($p<0.001$). Area under ROC curve (AUC) was 0.78, and cut-off value 7.35μU/mL for FI was obtained (sensitivity: 0.69; specificity: 0.77).

Conclusions: People with increased FI are associated with a higher prevalence of MetS. The

For peer review only

For peer review only - http://bmjopen.bmj.com/site/about/guidelines.xhtml

proposed cut-off value of FI may act as a marker for increased risk of MetS in middle-aged and elderly non-diabetic adults.

Keywords: metabolic syndrome, insulin resistance, fasting insulin level

Strengths and limitations of this study:

- This is the first study to explore the relationship between fasting insulin and MetS in the middle-aged and elderly populations of non-diabetic Asian people.
- We offer an easily measured biomarker to identify Chinese middle-aged and elderly with MetS.
- We adjusted many confounding factors to make the results more reliable.
- The recall bias from self-reported lifestyle behaviors is unavoidable.
- The causal relationship between fasting insulin level and MetS cannot be demonstrated in our cross-sectional study.

For peer review only

INTRODUCTION

Metabolic syndrome (MetS) is associated with a cluster of unhealthy metabolic risk factors, including abdominal obesity (excess body fat around the waist), glucose intolerance, pre-morbid hypertension, and dyslipidemia[1-3]. A number of studies have reported that MetS increases the risk of type 2 diabetes mellitus (T2DM), cardiovascular disease (CVD) and other non-communicable diseases (NCDs)[4-7]. The rising prevalence of MetS has created a disturbing challenge to personal health[8-12].

Insulin resistance has long been associated with MetS[13-16]. Basal insulin represents 45%-50% of daily insulin[17 18]. And the FI level approximates basal insulin[18 19]Studies have shown that FI levels may be associated with the prevalence of MetS, which may be due to its representativeness of insulin resistance[20 21]. A study has even shown that elevated FI levels may predict the future incidence of MetS[19]. According to DeBoer et al. different ethnics may reach the criteria for MetS at different stages of insulin resistance[22]. If insulin resistance is the foundation of MetS[14 15], and FI represents insulin resistance with an area under curve(AUC)[95% confidence interval(CI)] of 0.995[0.993-0.996] [20],a high FI level may be able to caution the physician for susceptibility to metabolic diseases and hence cardiovascular risks[23]. We therefore determined the relationships between fasting plasma insulin and MetS in middle-aged and elderly populations. In addition, we also determined a cut-off value for FI as a biomarker for the prevalence of MetS. By determining a cutoff value for FI, we could educate patients at risk for MetS and recommend lifestyle modification at an earlier stage.

METHODS

Study subjects

This was an observational and cross-sectional study conducted at Linkou Chang Gung Memorial Hospital in Taoyuan County, Taiwan between January and October 2014. The

For peer review only - http://bmjopen.bmj.com/site/about/guidelines.xhtml

inclusion criteria included residents 50-90 years old and living in Guishan township. 619 residents were eligible for the study. A total of 400 residents agreed to participate in our health exam. Subjects were excluded if they had diabetes. 79 participants with diabetes mellitus were excluded. A total of 321 participants (111 males and 210 females) were ultimately enrolled for analysis. This study was approved by the Institutional Review Board of the hospital and written inform consent was obtained from all of the participants before enrollment.

Data collection

We obtained exercise (exercising ≥3 times a week or not) and dietary habits (vegetarian or not) from self-administered questionnaires, which also included smoking (current smoker or not) and marital status (currently married or not). Anthropometric data, such as height, weight, waist circumference (WC), and blood pressure were also recorded. The subjects were dressed in light clothing without shoes for weight and height measurements. The BMI was calculated as the weight in kilograms (kg) divided by the height in meters squared (m^2). Waist circumference was measured midway between the inferior margin of the lowest rib and the iliac crest in the horizontal plane while in an upright position. Systolic blood pressure (SBP) and diastolic blood pressure (DBP) were checked at least 2 times after 5 min of rest while seated. Fasting insulin levels, lipid profile, and fasting glucose were obtained by blood sampling after a 10-h overnight fast. Blood samples were analyzed in the central laboratory of Linkou Chang Gung Memorial Hospital for fasting plasma glucose (FPG), serum total cholesterol (TC), low-density lipoprotein-cholesterol (LDL-C), high-density lipoprotein-cholesterol (HDL-C), serum triglycerides (TG), and fasting insulin levels. Serum insulin levels were determined with an ARCHITECT Insulin assay (Abbott Laboratories, IL, USA). Insulin was measured with a chemiluminescent microparticle immunessay (CMIA). The intra-assay variation and inter-assay variations were less than 2.7%. The ARCHITECT Insulin

For peer review only

For peer review only - http://bmjopen.bmj.com/site/about/guidelines.xhtml

assay has a sensitivity of ≤1.0μU/ml.

Defining MetS

MetS was defined by at least 3 of 5 metabolic syndrome diagnostic components (MetS-DCs), according to The Third Report of the National Cholesterol Education Program Expert Panel (NCEP) on Adult Treatment Panel (ATP III) Asian diagnostic criteria[24]. The five MetS-DCs were as follows: 1) SBP ≥ 130 mmHg and/or DBP ≥ 85 mmHg, or the use of anti-hypertensive drugs; 2) decreased serum HDL-C concentration < 40 mg/dl in men and <50 mg/dL in women, or treatment for dyslipidemia; 3) TG concentration ≥ 150 mg/dl, or on medication for hypertriglyceridemia; 4) hyperglycemia: fasting plasma glucose level ≥ 100 mg/dl; and 5) abdominal WC≥ 90 cm in men or ≥ 80 cm in women.

Statistical Analysis

Subjects were classified in one of three groups according to serum insulin level tertiles as the low, middle, and high insulin groups. Clinical characteristics were expressed as the mean ± standard deviation (SD) for continuous variables and number (%) for categorical variables. One-way analysis of variance (ANOVA) or a Chi-square test was used to determine p-values for continuous or categorical variables, respectively. Pearson's correlation was performed for each MetS-DC in relation to fasting insulin levels. The Cochran-Armitage trend test was used to evaluate the increasing prevalence of MetS-DCs as a function of insulin level tertile. The low fasting insulin group was designated as the reference group to calculate the ORs of the prevalence of MetS in the middle and high fasting insulin groups using multivariate logistic regression. Confounded variables present as an obstacle to valid inference in MetS studies. Hypertension and dyslipidemia are both common chronic conditions that affect a large proportion of the general adult population. Previous studies determining the association of FI and MetS also adjusted MetS-DCs[19]. Results of the adjusted model provide valid inference among MetS and insulin levels. A receiver operating characteristic curve (ROC) curve was

For peer review only - http://bmjopen.bmj.com/site/about/guidelines.xhtml

For peer review only

created for fasting insulin as a predictor of MetS. The area under the ROC curve (AUC) was analyzed, and the optimized cut-off point for fasting insulin, sensitivity, and specificity were acquired using the maximal Youden's Index. We used SPSS (version 23.0 for windows), to perform the statistical analysis. Statistical significance was set at a p-value < 0.05.

RESULTS

A total of 321 individuals, 111 men (34.6%) and 210 (65.4%) women, with a mean age of 63.91±8.32 years, were enrolled in this study. There were 90 study participants (28%) who met the diagnosis of MetS.

Table 1 shows the characteristics of the study population, which was divided based on the fasting insulin level in µU/mL. There were no statistically significant differences in age or gender between the low, middle, and high insulin level groups, while differences did exist with respect to WC, SBP, HDL-C, TG, and the proportion with MetS. Table 2 further shows the correlation between the fasting insulin level and all MetS-DCs, even after adjusting for age. Fasting insulin was positively correlated with SBP, WC, FPG, and TG, and negatively correlated with HDL-C, as shown in Table 2. Table 3 shows the prevalence of MetS-DCs (hypertension, hyperglycemia, dyslipidemia, and central obesity) according to the insulin level tertiles. The prevalence of MetS-DCs increased as the fasting insulin level increased, as shown by significant p values (Cochran-Armitage trend test). Figure 1 shows that the low insulin level group had a 10% prevalence of MetS, the middle insulin level group had a 21.5% prevalence of MetS, and the high insulin level group had a 53.8% of MetS (p<0.0001 [Cochran-Armitage trend test]), suggesting that the prevalence of MetS increased with an increase in fasting insulin levels.

For peer review only - http://bmjopen.bmj.com/site/about/guidelines.xhtml

Table 1 General characteristics of the study population based on insulin levels.

	Insulin levels				
	**Total**	**Low**	**Middle**	**High**	**p value**
**Variables**	**n=321**	**n=110 (≦4.8)**	**n=107 (4.9-7.8)**	**n=104 (≧7.9)**	
Age (year)	63.91 ±8.32	64.23 ±8.32	64.47 ±8.67	63.01 ±7.93	0.40
BMI (kg/m^2)	24.36 ±3.53	22.41 ±3.14	24.41 ±2.73	26.37 ±3.54	<0.001
Waist circumference (cm)	84.23 ±9.51	79.69 ±7.57	83.78 ±8.63	89.51 ±9.65	<0.001
SBP (mmHg)	129.02 ±16.59	123.69 ±17.36	129.52 ±14.51	134.13 ±16.20	<0.001
DBP (mmHg)	77.01 ±10.90	75.43 ±11.80	76.92 ±10.03	78.79 ±10.60	0.08
ALT (U/L)	21.74 ±11.06	18.94 ±7.81	20.33 ±9.25	26.15 ±14.05	<0.001
Creatinine (mg/dL)	0.76 ±0.44	0.69 ±0.17	0.85 ±0.66	0.75 ±0.34	0.03
FPG (mg/dL)	89.10 ±9.93	85.29 ±9.11	89.18 ±8.52	93.05 ±10.60	<0.001
HDL-C (mg/dL)	55.70 ±14.05	60.93 ±14.85	55.59 ±13.17	50.28 ±11.94	<0.001
Insulin (µU/mL)	7.10 ±4.14	3.60 ±0.94	6.21 ±0.86	11.72 ±4.02	<0.001
LDL-C (mg/dL)	121.48 ±32.05	118.90 ±34.55	126.03 ±31.01	119.53 ±30.10	0.20
T-cholesterol (mg/dL)	200.61 ±35.20	198.85 ±36.98	203.81 ±35.12	119.18 ±33.43	0.52
TG (mg/dL)	117.34 ±60.61	95.39 ±45.13	111.04 ±49.83	147.05 ±72.48	<0.001
Current smoking, n(%)	34 (10.6)	14 (12.7)	11 (10.3)	9 (8.7)	0.62
Marital status (single), n(%)	54 (16.8)	22 (20.0)	14 (13.1)	18 (17.3)	0.39
Men, n(%)	111 (34.6)	41 (37.3)	39 (36.4)	31 (29.8)	0.46
Regular exercise, n(%)	264 (82.2)	92 (83.6)	96 (89.7)	76 (73.1)	0.01
Vegetarian, n(%)	20 (6.2)	7 (6.4)	7 (6.5)	6 (5.8)	0.97
HTN, n(%)	150 (46.7)	43 (39.1)	47 (43.9)	60 (57.7)	0.02
Hyperlipidemia, n(%)	204 (63.6)	58 (52.7)	69 (64.5)	77 (74.0)	0.005
Metabolic syndrome, n(%)	90 (28.0)	11 (10.0)	23 (21.5)	56 (53.8)	<0.001

Notes: Ranges of FI levels of different tertile groups are shown in brackets at the top of the table, units in µU/mL.

Clinical characteristics are expressed as the mean±SD for continuous variables and n(%) for categorical variables. P-values were derived from one-way analysis of variance (ANOVA) for

For peer review only

continuous variables and chi-square test for categorical variables.

Abbreviations: BMI, body mass index; SBP, systolic blood pressure; DBP, diastolic blood pressure; ALT, alanine aminotransferase; FPG, fasting plasma glucose; HDL-C, high-density lipoprotein cholesterol; LDL-C, low-density lipoprotein cholesterol; TG, triglyceride; HTN, hypertension.

When designating the low insulin level group as the reference, the middle and high insulin level groups had an OR of 2.46 (p=0.02) and 10.50 (p<0.001) for MetS, respectively. After adjusting age, gender, and BMI, the middle and high insulin level groups had an OR of 1.71 (p= 0.20) and 5.63 (p <0.001) for MetS, respectively. After adjusting age, gender, BMI, smoking, hypertension and dyslipidemia, the middle and high insulin level groups still had an OR of 1.51 (p=0.35) and 5.04 (p<0.001; Table 4) for MetS, respectively. There was no significant difference between the middle and low tertile groups, but a significant difference between the high and low tertile groups, even after adjusting for the above risk factors. Based on this data, the high insulin level group had a 5-fold times risk for MetS compared to the low insulin level group.

Figure 2 shows the ROC curve of the insulin level as a predictor for MetS. The AUC was 0.78. The optimized cut-off value for insulin was shown to be 7.35 µU/mL, with a sensitivity of 0.69 and a specificity of 0.77.

For peer review only

For peer review only - http://bmjopen.bmj.com/site/about/guidelines.xhtml

DISCUSSION

In this community-based study, we investigated fasting serum insulin levels in association with the prevalence of MetS in non-diabetic middle-aged and elderly Taiwanese adults. In our study, the prevalence of MetS in the relatively healthy middle age-to-elderly population was 28%, which is similar to the 29.75% findings reported by Li et al.[25] Among middle-aged and elderly populations in Taiwan. Looking at the three ~~groups as a function of~~ fasting insulin tertiles, there was a rising proportion of MetS as the fasting insulin level increased, also shown in previous studies[20 26 27]. This finding not only applied to MetS, but to MetS-DCs as well. The WC, SBP, TG, and FPG levels were the lowest in the low fasting insulin group and highest in the high fasting insulin group; the converse applied to HDL and vice versa (Table 1). This finding led us to speculate that an association exists between fasting insulin levels and MetS-DCs. We found a statistically significant correlation between fasting insulin levels and each MetS-DC, even after adjusting for age (Table 2), in accordance to a study in adolescents [22]. A trend existed between the fasting insulin level and the prevalence of all five MetS-DCs (Table 3). We thus wanted to know if the trend applied to the prevalence of MetS. Figure 1 shows an apparent increase in the prevalence of MetS as the fasting insulin level increased. The trend was confirmed by the Cochran-Armitage trend test ($p<0.0001$).

For peer review only - http://bmjopen.bmj.com/site/about/guidelines.xhtml

Table 2 Pearson's correlation coefficients for each component of metabolic syndrome and age in relation to insulin levels.

Variables	Insulin(n=321)			
	Unadjusted		Adjusted for age	
	Pearson's coefficient	p value	Pearson's coefficient	p value
Age (year)	-0.04	0.50	NA	NA
SBP (mmHg)	0.21	<0.001	0.22	<0.001
DBP (mmHg)	0.11	0.05	0.10	0.07
Waist circumference (cm)	0.43	<0.001	0.44	<0.001
FPG (mg/dL)	0.38	<0.001	0.39	<0.001
HDL-C (mg/dL)	-0.37	<0.001	-0.37	<0.001
TG (mg/dL)	0.37	<0.001	0.37	<0.001

Abbreviations: SBP, systolic blood pressure; DBP, diastolic blood pressure; FPG, fasting plasma glucose; HDL-C, high-density lipoprotein cholesterol; TG, triglyceride.

For peer review only

206 **Table 3** Prevalence of components of metabolic syndrome based on insulin levels.

Components	Low (n=110) N(%)	Middle (n=107) N(%)	High (n=104) N(%)	p value for Cochran-Armitage trend test
High blood pressure[a]	56(50.9)	63(58.9)	78(75)	0.0003
High blood glucose[b]	8(7.3)	10(9.3)	25(24.0)	0.0004
Low HDL-C[c]	15(13.6)	19(17.8)	43(41.3)	<0.0001
High TG[d]	17(15.5)	27(25.2)	42(40.4)	<0.0001
Central obesity[e]	34(30.9)	57(53.3)	82(78.8)	<0.0001

207 **Note:**

208 [a] SBP≧130 mmHg or DBP≧85 mmHg, or self-reported hypertension

209 [b] Fasting blood glucose ≥100 mg/dL or self-reported diabetes mellitus

210 [c] HDL-C<40 mg/dL in men or <50 mg/dL in women

211 [d] TG≥150 mg/dL

212 [e] Waist circumference ≥90 cm in men or ≥80 cm in women

213 **Abbreviations:** HDL-C, high-density lipoprotein-cholesterol; TG, triglyceride; SBP, systolic
214 blood pressure; DBP, diastolic blood pressure.

For peer review only

After adjusting for gender, age, BMI, smoking status, hypertension, and dyslipidemia, the middle-aged and elderly populations in the high fasting insulin group were at significant risk for developing MetS (OR = 5.04, 95% CI =2.15-11.81; P＜0.01: Table 4). This conclusion is consistent with previous findings[20 27 28]. Not only is fasting insulin an independent risk factor for MetS, but in the cohort study of Sung et al., it was also reported that elevated fasting insulin predicted the future incidence of MetS[19]. One possible explanation might be the relationship between the fasting insulin level and insulin resistance[20 21], which has a fundamental role in MetS[29 30]. Although the mechanism by which fasting insulin may represent insulin resistance was not investigated in the present study, a number of studies have shown that fasting insulin is a suitable surrogate marker for insulin resistance[21 31-34], calculated by the fasting insulin resistance index (FIRI) or homeostasis model assessment of insulin resistance (HOMA-IR). Based on these equations, a higher fasting insulin level can contribute to insulin resistance in patients with impaired fasting glucose, but may be an inappropriate marker in diabetics with poor glycemic control. This finding may be the reason that a higher fasting insulin level increases the risk of MetS in non-diabetic populations. It has been reported that the fasting insulin level is highly associated with MetS, but a cutoff value was not established [20]. Based on our search of the literature, there is no widely accepted reference range for fasting insulin. A reference range for fasting insulin of 1.57–16.32 µU/mL has been proposed in Chinese men, but the reference range varies between different ethnicities and genders [35]. A fasting insulin level > 9µU/mL has been reported to identify 80% of patients with pre-diabetes [36]. In our study, the AUC for fasting insulin as an indication of risk for MetS was 0.78, similar to another study's AUC of 0.77[20]. Our proposed cut-off value for fasting insulin was 7.35 µU/mL (sensitivity=0.69 and specificity=0.77). Because MetS is a state of pre-diabetes[30], our cutoff value < 9µU/mL is reasonable. Our proposed cut-off value of 7.35 µU/mL is between the mean fasting insulin levels of the

For peer review only - http://bmjopen.bmj.com/site/about/guidelines.xhtml

middle and high tertile groups. This may be reasonable because the middle and low tertile groups did not show a statistically significant difference in OR for MetS after adjusting for confounding factors.

For peer review only

For peer review only - http://bmjopen.bmj.com/site/about/guidelines.xhtml

Table 4 Association between insulin levels and metabolic syndrome.

Variables	Model 1			Model 2			Model 3		
	**OR**	**(95% CI)**	**p value**	**OR**	**(95% CI)**	**p value**	**OR**	**(95% CI)**	**p value**
Low	1.00	—	—	1.00	—	—	1.00	—	—
Middle	2.46	(1.14-5.35)	0.02	1.71	(0.76-3.85)	0.20	1.51	(0.64-3.57)	0.35
High	10.50	(5.05-21.84)	<0.001	5.63	(2.53-12.53)	<0.001	5.04	(2.15-11.81)	<0.001
p value for trend			<0.001			<0.001			<0.001

Model 1: unadjusted

Model 2: adjusted for gender, age, and BMI

Model 3: adjusted for factors in model 2 plus smoking, HTN, and hyperlipidemia

243 **Abbreviations:** BMI, body mass index; HTN, hypertension; OR, odds ratio; CI, confidence
244 interval.

For peer review only - http://bmjopen.bmj.com/site/about/guidelines.xhtml

245 **Table 5** Studies of Association Between Fasting Insulin and Metabolic Syndrome.

Authors	Study Year	Study Population	% MetS	Fasting insulin	Risk of MetS	Main Finding	Reference
Saravia G et al 2015	2009-2010 Cross sectional	3200 Non-diabetic males in Spain	23%	Highest tertile (≥6.13) vs lowest (≤3.80)µU/mL	OR (95%CI) 11.36 (8.65-15.13) for MetS	Per each 10 pmol/L (1.4 uU/mL) increase in insulin, the odds for metabolic syndrome increased by 1.43 (95%CI: 1.38, 1.49)	[20]
Rutter MK et al 2014	(1991-1995) to (1998-2001) 7 year Prospective	2616 non-diabetic adults in Europe	-	1-quintile change in fasting insulin (pmol/L)	mean (95% CI) 0.12 (0.10–0.15) [MetS trait score 7 year change]	Change in metabolic trait clustering was significantly associated with baseline levels and changes in fasting insulin.	[38]
Sung KC et al 2011	2003-2008 5 year cohort	2350 non-MetS in Korea	8.5% (incidence)	Highest quartile (≥8.98) vs lowest (≤6.01) IU/ml	OR (95% CI) of developing MS 5.1 (3.1-8.2)	The highest quartile of the insulin levels had more than a 5 times greater risk of developing MS compared to the subjects in the lowest quartile.	[19]
Kanda H et al 2011	2000,2001 Cross sectional	456 in Mongolia	6.4%	Highest tertile (≥10.33) vs lowest (≤6.72) mmol/L	Percentage of MetS 17.1% vs 4.6%	Fasting plasma insulin is associated with MetS in farmers, but not nomads among the Mongolian population in China.	[27]
STOPP-T2D PSG* 2008	2003 Cross sectional	1453 8th grade adolescents in the U.S.	9.5%	Highest quintile (≥39.1) vs lowest (≤17.0) µU/mL	OR (95%CI) 199.64(31.29-1273.7) for MetS	The highest insulin quintile were almost 200 times more likely to be classified with the metabolic syndrome than participants in the lowest quintile.	[28]
Adam FM et al 2006	2005 Cross sectional	128 overweight/ obese in Indonesia	68.8%	Mean fasting insulin levels15.68±7.85 vs 3.16±2.53 (uU/ml) with 5 components vs 1 component of MetS.		There is a strong linear increase in fasting insulin levels with an increase of the number of metabolic syndrome.	[29]

246 *STOPP-T2D PSG: Studies to Treat or Prevent Pediatric Type 2 Diabetes Prevention Study Group

247

For peer review only - http://bmjopen.bmj.com/site/about/guidelines.xhtml

Our findings may have an impact on health screening policies in people older than middle-age. The diagnosis criteria of MetS differs according to gender, ethnicity and even age. A study shows that certain ethnic groups do not meet current criteria of MetS until they have reached a more advanced degree of insulin resistance[22]. Elevated FI, however, may act as a marker to alert physicians on the risk of MetS in this individual. Given the fact that elevated FI is not only associated with a greater risk for developing MetS [19 37] but is also associated with a greater number of cardiometabolic risk factors[23], lifestyle modification should be considered when the FI level is > 7.35 μU/mL, due to the amelioration of metabolic abnormalities with diet or exercise interventions[38-40].

Some limitations in our study merit consideration. First, the principal limitation relevant to the interpretation of our results was the use of a cross-sectional design, thus a causal relationship between the FI level and MetS cannot be inferred. Second, the sample size in our study was relatively small (n=321[the power was not calculated]) and the participants were recruited from a regional community. The participants could therefore only be distributed into three groups and the results cannot be generalized to other ethnicities. Third, though males tend to have a lower participation rate in studies [41], there may still have been a selection bias due to the higher participation of women than men in our study. Fourth, according to our cutoff value, there is a false positive rate of 23% and a false negative rate of 31%. The false positive rate can be accepted due to our proposed intervention, early lifestyle modification, is less likely to do harm to the population. The false negative rate should be taken into consideration of the physician when applying this data. Furthermore, even though we used a standardized questionnaire, recall and reporting bias are unavoidable for self-reported data. Finally, we did not ask participants to sleep adequately or to avoid vigorous exercise the day before blood testing, which could affect the accuracy of the fasting serum insulin level.

Our study also has strengths. First, our participants were recruited during a community

For peer review only - http://bmjopen.bmj.com/site/about/guidelines.xhtml

health examination and represent a relatively healthy population. The effects of important confounders, including ethnicity, residential area, and environmental factors, were minimized. Second, we used standardized laboratory examination protocols and anthropometric measurements. Third, while evaluating the association between MetS and the fasting insulin level, we excluded diabetic patients to avoid the effect of anti-diabetic medications on the fasting insulin level. Lastly, due to the trend of world aging, our study aimed for middle-aged and elderly populations. And given the differences of FI levels in different ethnic groups [22], our study contributes to the Taiwanese population. Studies from all around the world indicate the relationship of FI and MetS (table 5), though few physicians have applied to their practice.

In the future, we will continue to follow this community and record the development of newly diagnosed MetS. Counseling of lifestyle modification for residents with elevated FI will also be our topic of interest hereon.

CONCLUSIONS

This study provides a convenient method to identify the risk of MetS by testing FI levels in the non-diabetic populations. We suggest a FI cut-off value of 7.35 μU/mL to start lifestyle modificaitons in the middle-aged and elderly non-diabetic population. We believe that the cut-off value can be of use to physicians, which cautions the risk of MetS. Providing medical counseling to patients with a FI level >7.35 μU/mL should result in long-term health benefits. But further studies may be needed for this conclusion.

For peer review only

Contributors YHC and YCL were involved in writing of the manuscript. YCT, MCL, HHC and WCY conceived and supervised the study. IST provided statistical advice. JYC contributed conceived, designed and performed the experiments, collected and analyzed the data, revising it critically for important intellectual content and final approval of the version to be submitted.

Funding This work was supported by Chang Gung Memorial Hospital (CORPG3C0171, CORPG3C0172, CZRPG3C0053).

Competing interests None declared.

Ethics approval The study was approved by Chang-Gung Medical Foundation Institutional Review Board (102-2304B), and written informed consent was given by all the participants before enrollment.

Data sharing statement No additional data are available.

REFERENCES

1. Kaur J. A comprehensive review on metabolic syndrome. *Cardiol Res Pract* 2014;2014:943162. doi: 10.1155/2014/943162

2. Nakao YM, Miyawaki T, Yasuno S, et al. Intra-abdominal fat area is a predictor for new onset of individual components of metabolic syndrome: MEtabolic syndRome and abdominaL ObesiTy (MERLOT study). *Proc Jpn Acad Ser B Phys Biol Sci* 2012;88(8):454-61.

3. Popa S, Mota M, Popa A, et al. Prevalence of overweight/obesity, abdominal obesity and metabolic syndrome and atypical cardiometabolic phenotypes in the adult Romanian population: PREDATORR study. *J Endocrinol Invest* 2016;39(9):1045-53. doi: 10.1007/s40618-016-0470-4

4. Li W-C, Chen J-Y, Lin C-H, et al. Reevaluating the Diagnostic Criteria for Metabolic

For peer review only

Syndrome in the Taiwanese Population. *Journal of the American College of Nutrition* 2011;30(4):241-47.

5. Dragsbaek K, Neergaard JS, Laursen JM, et al. Metabolic syndrome and subsequent risk of type 2 diabetes and cardiovascular disease in elderly women: Challenging the current definition. *Medicine (Baltimore)* 2016;95(36):e4806. doi: 10.1097/MD.0000000000004806

6. Meifang Y, Xue S, Jue H, et al. [Metabolic syndrome increases Framingham risk score of patients with type 2 diabetes mellitus]. *Zhejiang Da Xue Xue Bao Yi Xue Ban* 2016;45(3):268-74.

7. Oguoma VM, Nwose EU, Skinner TC, et al. Association between metabolic syndrome and 10-year risk of developing cardiovascular disease in a Nigerian population. *Int Health* 2016 doi: 10.1093/inthealth/ihw013

8. Desroches S, Lamarche B. The evolving definitions and increasing prevalence of the metabolic syndrome. *Appl Physiol Nutr Metab* 2007;32(1):23-32. doi: 10.1139/h06-095

9. Ford ES, Giles WH, Mokdad AH. Increasing prevalence of the metabolic syndrome among u.s. Adults. *Diabetes Care* 2004;27(10):2444-9.

10. Hu G, Lindstrom J, Jousilahti P, et al. The increasing prevalence of metabolic syndrome among Finnish men and women over a decade. *J Clin Endocrinol Metab* 2008;93(3):832-6. doi: 10.1210/jc.2007-1883

11. Lim S, Shin H, Song JH, et al. Increasing prevalence of metabolic syndrome in Korea: the Korean National Health and Nutrition Examination Survey for 1998-2007. *Diabetes Care* 2011;34(6):1323-8. doi: 10.2337/dc10-2109

12. Liu M, Wang J, Jiang B, et al. Increasing Prevalence of Metabolic Syndrome in a Chinese Elderly Population: 2001-2010. *PLoS One* 2013;8(6):e66233. doi: 10.1371/journal.pone.0066233

For peer review only

For peer review only - http://bmjopen.bmj.com/site/about/guidelines.xhtml

13. Grundy SM, Brewer HB, Jr., Cleeman JI, et al. Definition of metabolic syndrome: Report of the National Heart, Lung, and Blood Institute/American Heart Association conference on scientific issues related to definition. *Circulation* 2004;109(3):433-8. doi: 10.1161/01.CIR.0000111245.75752.C6

14. Lann D, LeRoith D. Insulin resistance as the underlying cause for the metabolic syndrome. *Med Clin North Am* 2007;91(6):1063-77, viii. doi: 10.1016/j.mcna.2007.06.012

15. Gallagher EJ, Leroith D, Karnieli E. Insulin resistance in obesity as the underlying cause for the metabolic syndrome. *Mt Sinai J Med* 2010;77(5):511-23. doi: 10.1002/msj.20212

16. Dontsov AV, Vasil'eva LV. [Insulin Resistance Associated with Metabolic Syndrome as an Indicator of Cardiovascular Risk]. *Klin Med (Mosk)* 2016;94(3):189-93.

17. Polonsky KS, Given BD, Van Cauter E. Twenty-four-hour profiles and pulsatile patterns of insulin secretion in normal and obese subjects. *J Clin Invest* 1988;81(2):442-8. doi: 10.1172/JCI113339

18. Wilcox G. Insulin and insulin resistance. *Clin Biochem Rev* 2005;26(2):19-39.

19. Sung KC, Seo MH, Rhee EJ, et al. Elevated fasting insulin predicts the future incidence of metabolic syndrome: a 5-year follow-up study. *Cardiovasc Diabetol* 2011;10:108. doi: 10.1186/1475-2840-10-108

20. Saravia G, Civeira F, Hurtado-Roca Y, et al. Glycated Hemoglobin, Fasting Insulin and the Metabolic Syndrome in Males. Cross-Sectional Analyses of the Aragon Workers' Health Study Baseline. *PLoS One* 2015;10(8):e0132244. doi: 10.1371/journal.pone.0132244

21. ter Horst KW, Gilijamse PW, Koopman KE, et al. Insulin resistance in obesity can be reliably identified from fasting plasma insulin. *Int J Obes (Lond)* 2015;39(12):1703-9. doi: 10.1038/ijo.2015.125 [published Online First: 2015/07/15]

22. DeBoer MD, Dong L, Gurka MJ. Racial/ethnic and sex differences in the ability of

For peer review only

For peer review only - http://bmjopen.bmj.com/site/about/guidelines.xhtml

metabolic syndrome criteria to predict elevations in fasting insulin levels in adolescents. *J Pediatr* 2011;159(6):975-81 e3. doi: 10.1016/j.jpeds.2011.05.023

23. Pataky Z, Golay A, Laville M, et al. Fasting insulin at baseline influences the number of cardiometabolic risk factors and R-R interval at 3years in a healthy population: the RISC Study. *Diabetes Metab* 2013;39(4):330-6. doi: 10.1016/j.diabet.2013.05.008 [published Online First: 2013/07/24]

24. Tan CE, Ma S, Wai D, et al. Can we apply the National Cholesterol Education Program Adult Treatment Panel definition of the metabolic syndrome to Asians? *Diabetes Care* 2004;27(5):1182-6.

25. Li Z, Yang X, Yang J, et al. The Cohort Study on Prediction of Incidence of All-Cause Mortality by Metabolic Syndrome. *PLoS One* 2016;11(5):e0154990. doi: 10.1371/journal.pone.0154990

26. Kanda H, Wang P, Okamura T, et al. Fasting plasma insulin is associated with metabolic syndrome in farmers but not in nomads among the Mongolian population, China. *J Atheroscler Thromb* 2011;18(4):291-7.

27. Prevalence of the metabolic syndrome among a racially/ethnically diverse group of U.S. eighth-grade adolescents and associations with fasting insulin and homeostasis model assessment of insulin resistance levels. *Diabetes Care* 2008;31(10):2020-5. doi: 10.2337/dc08-0411 [published Online First: 2008/07/02]

28. Adam FM, Nara MG, Adam JM. Fasting insulin, adiponectin, hs-CRP levels, and the components of metabolic syndrome. *Acta Med Indones* 2006;38(4):179-84.

29. Saltiel AR. Series introduction: the molecular and physiological basis of insulin resistance: emerging implications for metabolic and cardiovascular diseases. *J Clin Invest* 2000;106(2):163-4. doi: 10.1172/JCI10533

30. Mayans L. Metabolic Syndrome: Insulin Resistance and Prediabetes. *FP Essent*

For peer review only

2015;435:11-6.
31. Duncan MH, Singh BM, Wise PH, et al. A simple measure of insulin resistance. *Lancet* 1995;346(8967):120-1.
32. Abbasi F, Reaven GM. Evaluation of the quantitative insulin sensitivity check index as an estimate of insulin sensitivity in humans. *Metabolism* 2002;51(2):235-7.
33. Kim SH, Abbasi F, Reaven GM. Impact of degree of obesity on surrogate estimates of insulin resistance. *Diabetes Care* 2004;27(8):1998-2002.
34. Tang Q, Li X, Song P, et al. Optimal cut-off values for the homeostasis model assessment of insulin resistance (HOMA-IR) and pre-diabetes screening: Developments in research and prospects for the future. *Drug Discov Ther* 2015;9(6):380-5. doi: 10.5582/ddt.2015.01207
35. Li S, Huang S, Mo ZN, et al. Generating a reference interval for fasting serum insulin in healthy nondiabetic adult Chinese men. *Singapore Med J* 2012;53(12):821-5.
36. Johnson JL, Duick DS, Chui MA, et al. Identifying prediabetes using fasting insulin levels. *Endocr Pract* 2010;16(1):47-52. doi: 10.4158/EP09031.OR
37. Rutter MK, Sullivan LM, Fox CS, et al. Baseline levels, and changes over time in body mass index and fasting insulin, and their relationship to change in metabolic trait clustering. *Metab Syndr Relat Disord* 2014;12(7):372-80. doi: 10.1089/met.2013.0148
38. Sacks FM, Bray GA, Carey VJ, et al. Comparison of weight-loss diets with different compositions of fat, protein, and carbohydrates. *N Engl J Med* 2009;360(9):859-73. doi: 10.1056/NEJMoa0804748
39. Vissers D, Hens W, Taeymans J, et al. The effect of exercise on visceral adipose tissue in overweight adults: a systematic review and meta-analysis. *PLoS One* 2013;8(2):e56415. doi: 10.1371/journal.pone.0056415
40. Hinderliter AL, Babyak MA, Sherwood A, et al. The DASH diet and insulin sensitivity.

For peer review only

For peer review only - http://bmjopen.bmj.com/site/about/guidelines.xhtml

Curr Hypertens Rep 2011;13(1):67-73. doi: 10.1007/s11906-010-0168-5

41. Galea S, Tracy M. Participation rates in epidemiologic studies. *Ann Epidemiol* 2007;17(9):643-53. doi: 10.1016/j.annepidem.2007.03.013 [published Online First: 2007/06/08]

Figure Legends

Figure 1. Prevalence of metabolic syndrome based on insulin levels. A linear increasing trend across insulin tertiles.

Figure 2. ROC curve for insulin as a predictor of metabolic syndrome.

For peer review only

For peer review only - http://bmjopen.bmj.com/site/about/guidelines.xhtml

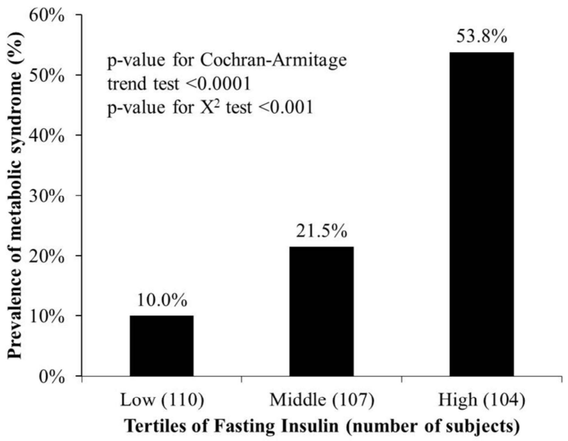

Figure 1 Prevalence of metabolic syndrome based on insulin levels. A linear increasing trend across insulin tertiles.

Figure 1. Prevalence of metabolic syndrome based on insulin levels. A linear increasing trend across insulin tertiles.

297x209mm (300 x 300 DPI)

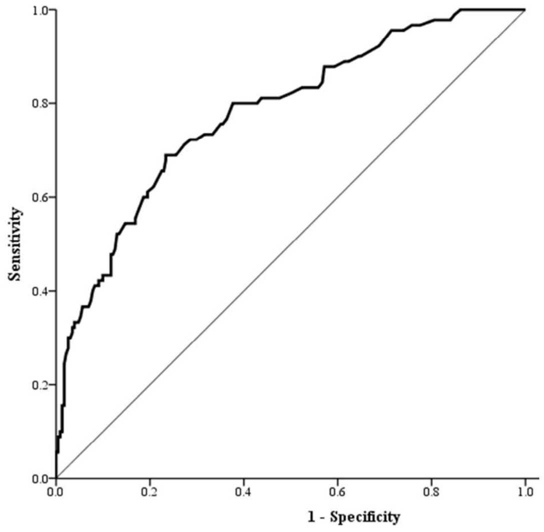

Figure 2 ROC curve for insulin as a predictor of metabolic syndrome.

Figure 2. ROC curve for insulin as a predictor of metabolic syndrome.

297x209mm (300 x 300 DPI)

For peer review only

STROBE 2007 (v4) Statement—Checklist of items that should be included in reports of *cross-sectional studies*

Section/Topic	Item #	Recommendation	Reported on page #
**Title and abstract**	1	(*a*) Indicate the study's design with a commonly used term in the title or the abstract	P2
		(*b*) Provide in the abstract an informative and balanced summary of what was done and what was found	P2
**Introduction**			
Background/rationale	2	Explain the scientific background and rationale for the investigation being reported	P4
Objectives	3	State specific objectives, including any prespecified hypotheses	P2, P4
**Methods**			
Study design	4	Present key elements of study design early in the paper	P4
Setting	5	Describe the setting, locations, and relevant dates, including periods of recruitment, exposure, follow-up, and data collection	P4, 5
Participants	6	(*a*) Give the eligibility criteria, and the sources and methods of selection of participants	P4
Variables	7	Clearly define all outcomes, exposures, predictors, potential confounders, and effect modifiers. Give diagnostic criteria, if applicable	P5
Data sources/ measurement	8*	For each variable of interest, give sources of data and details of methods of assessment (measurement). Describe comparability of assessment methods if there is more than one group	P5
Bias	9	Describe any efforts to address potential sources of bias	Nil
Study size	10	Explain how the study size was arrived at	Nil
Quantitative variables	11	Explain how quantitative variables were handled in the analyses. If applicable, describe which groupings were chosen and why	
Statistical methods	12	(*a*) Describe all statistical methods, including those used to control for confounding	P6
		(*b*) Describe any methods used to examine subgroups and interactions	P6
		(*c*) Explain how missing data were addressed	Nil
		(*d*) If applicable, describe analytical methods taking account of sampling strategy	Nil
		(*e*) Describe any sensitivity analyses	Nil
**Results**			

For peer review only

Participants	13*	(a) Report numbers of individuals at each stage of study—eg numbers potentially eligible, examined for eligibility, confirmed eligible, included in the study, completing follow-up, and analysed	Nil
		(b) Give reasons for non-participation at each stage	Nil
		(c) Consider use of a flow diagram	Nil
Descriptive data	14*	(a) Give characteristics of study participants (eg demographic, clinical, social) and information on exposures and potential confounders	P8
		(b) Indicate number of participants with missing data for each variable of interest	Nil
Outcome data	15*	Report numbers of outcome events or summary measures	P6-9
Main results	16	(*a*) Give unadjusted estimates and, if applicable, confounder-adjusted estimates and their precision (eg, 95% confidence interval). Make clear which confounders were adjusted for and why they were included	P7
		(*b*) Report category boundaries when continuous variables were categorized	P8
		(*c*) If relevant, consider translating estimates of relative risk into absolute risk for a meaningful time period	Nil
Other analyses	17	Report other analyses done—eg analyses of subgroups and interactions, and sensitivity analyses	Nil
**Discussion**			
Key results	18	Summarise key results with reference to study objectives	P7
Limitations	19	Discuss limitations of the study, taking into account sources of potential bias or imprecision. Discuss both direction and magnitude of any potential bias	P17
Interpretation	20	Give a cautious overall interpretation of results considering objectives, limitations, multiplicity of analyses, results from similar studies, and other relevant evidence	P17
Generalisability	21	Discuss the generalisability (external validity) of the study results	P18
**Other information**			
Funding	22	Give the source of funding and the role of the funders for the present study and, if applicable, for the original study on which the present article is based	P19

*Give information separately for cases and controls in case-control studies and, if applicable, for exposed and unexposed groups in cohort and cross-sectional studies.

Note: An Explanation and Elaboration article discusses each checklist item and gives methodological background and published examples of transparent reporting. The STROBE checklist is best used in conjunction with this article (freely available on the Web sites of PLoS Medicine at http://www.plosmedicine.org/, Annals of Internal Medicine at http://www.annals.org/, and Epidemiology at http://www.epidem.com/). Information on the STROBE Initiative is available at www.strobe-statement.org.

For peer review only

High fasting insulin levels are associated with metabolic syndrome in non-diabetic middle-aged and elderly populations: A community-based study in Taiwan

Journal:	*BMJ Open*
Manuscript ID	bmjopen-2017-016554.R2
Article Type:	Research
Date Submitted by the Author:	31-Oct-2017

Complete List of Authors:	Chen, Yun-Hung; Chang Gung Memorial Hospital Linkou Branch, Family Medicine<br>Lee, Yu-Chien; Chang Gung Memorial Hospital Linkou Branch, Family Medicine<br>Tsao, Yu-Chung; Chang Gung Memorial Hospital Linkou Branch, Family Medicine; Chang Gung Memorial Hospital Linkou Branch, Occupational Medicine<br>Lu, Mei-Chun; Chang Gung Memorial Hospital Linkou Branch, Family Medicine<br>Chuang, Hai-Hua; Chang Gung Memorial Hospital Taipei Branch, Family Medicine<br>Yeh, Wei-Chung; Chang Gung Memorial Hospital Linkou Branch, Family Medicine; Chang Gung University College of Medicine<br>Tzeng, I-Shiang; Taipei Tzu Chi General Hospital, Department of Research<br>Chen, Jau-Yuan; Chang Gung Memorial Hospital Linkou Branch, Family Medicine; Chang Gung University College of Medicine

**Primary Subject Heading**:	General practice / Family practice
Secondary Subject Heading:	General practice / Family practice, Geriatric medicine, Health policy
Keywords:	metabolic syndrome, insulin resistance, fasting insulin level

SCHOLARONE™ Manuscripts

- **Title**

High fasting insulin levels are associated with metabolic syndrome in non-diabetic middle-aged and elderly populations: A community-based study in Taiwan

- **Short title**

Increased fasting insulin levels in association with metabolic syndrome

- **Author names and affiliations**

Yun-Hung Chen[1], Yu-Chien Lee[1], Yu-Chung Tsao[1, 2, 3], Mei-Chun Lu[1], Hai-Hua Chuang[4], Wei-ChungYeh[1, 3], I-Shiang Tzeng[5], Jau-Yuan Chen[1, 3, *]

[1]Department of Family Medicine, Chang Gung Memorial Hospital, Linkou Branch, Taiwan

[2]Department of Occupational Medicine, Chang-Gung Memorial Hospital, Linkou Branch, Taiwan

[3]College of Medicine, Chang Gung University, Taoyuan, Taiwan

[4]Department of Family Medicine, Chang-Gung Memorial Hospital, Taipei Branch, Taiwan

[5]Department of Research, Taipei Tzu Chi Hospital, Buddhist Tzu Chi Medical Foundation, New Taipei city, Taiwan

*Corresponding author

E-mail: welins@cgmh.org.tw (J.-Y. Chen)

Postal address: No. 5, Fuxing St., Guishan Dist., Taoyuan City 333, Taiwan (R.O.C.)

Phone: +886-975362672

Fax: +886-3-3287715

- **Format**

Word count: 2500

Number of black and white figures: 2

Number of Tables: 5

Number of References: 37

For peer review only - http://bmjopen.bmj.com/site/about/guidelines.xhtml

ABSTRACT

Objectives: We determined the association between fasting insulin (FI) levels and metabolic syndrome (MetS), and suggested a value of FI which can help detect possible comorbidity of MetS in non-diabetic middle-aged and elderly adults.

Design: Cross-sectional observational study.

Setting: Community-based investigation in Guishan township of northern Taiwan.

Participants: Our study included adults aged 50 years and above during community health exams between January and October 2014. People with diabetes mellitus were excluded. A total of 321 people were enrolled.

Outcome measures: We divided participants according to tertiles of FI as low, medium, and high levels. Pearson correlation was assessed between insulin level and each of the diagnostic components of metabolic syndrome (MetS-DCs) with adjustment of age. The prevalence of MetS-DCs based on tertiles of FI were studied and analyzed by Cochran-Armitage trend test. The risk for prevalence of MetS in the middle, and high insulin group as compared to the low insulin group were assessed by multivariate logistic regression with adjustments for age, gender, smoking, body mass index (BMI), hypertension, and hyperlipidemia. Youden Index was performed for the optimized cut-off value.

Results: Our results showed positive correlation of FI level with systolic blood pressure, waist circumference, fasting plasma glucose and triglyceride levels, while negative correlation was shown with high-density lipoprotein ($p<0.001$). The prevalence of each MetS-DCs increased as a trend while FI levels increased ($p<0.001$). OR (95% CI) of MetS was 5.04 (2.15-11.81) for high insulin groups compared to the low insulin group after adjusting confounders ($p<0.001$). Area under receiver operating characteristic curve (ROC) curve (AUC) was 0.78, and cut-off value 7.35 μU/mL for FI was obtained (sensitivity: 0.69; specificity: 0.77).

For peer review only

For peer review only - http://bmjopen.bmj.com/site/about/guidelines.xhtml

Conclusions: Middle-aged and elderly non-diabetic people with increased FI are associated with a higher prevalence of MetS. Furthermore, FI is an independent risk factor of MetS.

Keywords: metabolic syndrome, insulin resistance, fasting insulin level

Strengths and limitations of this study:

- This is the first study to explore the relationship between fasting insulin and MetS in the middle-aged and elderly populations of non-diabetic Asian people.
- We offer a biomarker to identify middle-aged and elder non-diabetic Taiwanese with MetS.
- We adjusted many confounding factors to make the results more reliable.
- The recall bias from self-reported lifestyle behaviors is unavoidable.
- The causal relationship between fasting insulin level and MetS cannot be demonstrated in our cross-sectional study.

For peer review only

INTRODUCTION

Metabolic syndrome (MetS) is associated with a cluster of unhealthy metabolic risk factors, including abdominal obesity (excess body fat around the waist), glucose intolerance, pre-morbid hypertension, and dyslipidemia[1-3]. A number of studies have reported that MetS increases the risk of type 2 diabetes mellitus (T2DM), cardiovascular disease (CVD) and other non-communicable diseases (NCDs)[4-7]. The rising prevalence of MetS has created a disturbing challenge to personal health[8-12].

Insulin resistance has long been associated with MetS[13-16]. Basal insulin represents 45%-50% of daily insulin[17 18], and the FI level approximates basal insulin[18 19]. Studies have shown that FI levels are associated with the prevalence of MetS, which may be due to its representativeness of insulin resistance[20 21]. A study has even shown that elevated FI levels may predict the future incidence of MetS[19]. If insulin resistance is the foundation of MetS[14 15], and FI represents insulin resistance with an area under curve (AUC) [95% confidence interval (CI)] of 0.995[0.993-0.996] [20], a high FI level may be able to caution the physician for susceptibility to metabolic diseases and hence cardiovascular risks[22]. We therefore determined the relationships between FI and MetS in middle-aged and elderly populations. In addition, we also obtained a cut-off value for FI as a biomarker for the risk of MetS.

METHODS

Study subjects

This was an observational and cross-sectional study conducted at Linkou Chang Gung Memorial Hospital in Taoyuan County, Taiwan between January and October 2014. The inclusion criteria included residents 50-90 years old and living in Guishan township. 619 residents were eligible for the study. A total of 400 residents agreed to participate in our health exam. Subjects were excluded if they had diabetes. 79 participants with diabetes

For peer review only - http://bmjopen.bmj.com/site/about/guidelines.xhtml

mellitus were excluded. Diabetes mellitus was defined as any of the followings: 1. Previous diagnosis of diabetes mellitus; 2. Recent use of oral anti-hyperglycemic drugs or insulin; 3. Participants with fasting glucose≥126 mg/dl. A total of 321 participants (111 males and 210 females) were ultimately enrolled for analysis. This study was approved by the Institutional Review Board of the hospital and written informed consent was obtained from all of the participants before enrollment.

Data collection

We obtained exercise (exercising ≥3 times a week or not) and dietary habits (vegetarian or not) from self-administered questionnaires, which also included smoking (current smoker or not) and marital status (currently married or not). Anthropometric data, such as height, weight, waist circumference (WC), and blood pressure were also recorded. The subjects were dressed in light clothing without shoes for weight and height measurements. The BMI was calculated as the weight in kilograms (kg) divided by the height in meters squared (m^2). Waist circumference was measured midway between the inferior margin of the lowest rib and the iliac crest in the horizontal plane while in an upright position. Systolic blood pressure (SBP) and diastolic blood pressure (DBP) were checked at least 2 times after 5 min of rest while seated. FI levels, lipid profile, and fasting glucose were obtained by blood sampling after a 10-h overnight fast. Blood samples were analyzed in the central laboratory of Linkou Chang Gung Memorial Hospital for fasting plasma glucose (FPG), serum total cholesterol (TC), low-density lipoprotein-cholesterol (LDL-C), high-density lipoprotein-cholesterol (HDL-C), serum triglycerides (TG), and FI levels. Serum insulin levels were determined with an ARCHITECT Insulin assay (Abbott Laboratories, IL, USA). Insulin was measured with a chemiluminescent microparticle immunessay (CMIA). The intra-assay variation and inter-assay variations were less than 2.7%. The ARCHITECT Insulin assay has a sensitivity of ≤1.0μU/ml.

For peer review only - http://bmjopen.bmj.com/site/about/guidelines.xhtml

Defining MetS

MetS was defined by at least 3 of 5 metabolic syndrome diagnostic components (MetS-DCs), according to The Third Report of the National Cholesterol Education Program Expert Panel (NCEP) on Adult Treatment Panel (ATP III) Asian diagnostic criteria[23]. The five MetS-DCs were as follows: 1) SBP $\geq$ 130 mmHg and/or DBP $\geq$ 85 mmHg, or the use of anti-hypertensive drugs; 2) decreased serum HDL-C concentration $<$ 40 mg/dl in men and $<$50 mg/dL in women, or treatment for dyslipidemia; 3) TG concentration $\geq$ 150 mg/dl, or on medication for hypertriglyceridemia; 4) hyperglycemia: fasting plasma glucose level $\geq$ 100 mg/dl; and 5) abdominal WC$\geq$ 90 cm in men or $\geq$ 80 cm in women.

Statistical Analysis

Subjects were classified in one of three groups according to serum insulin level tertiles as the low, middle, and high insulin groups. Clinical characteristics were expressed as the mean $\pm$ standard deviation (SD) for continuous variables and number (%) for categorical variables. One-way analysis of variance (ANOVA) or a Chi-square test was used to determine p-values for continuous or categorical variables, respectively. Pearson's correlation was performed for each MetS-DC in relation to FI levels. The Cochran-Armitage trend test was used to evaluate the increasing prevalence of MetS-DCs as a function of insulin level tertile. The low FI group was designated as the reference group to calculate the ORs of the prevalence of MetS in the middle and high FI groups using multivariate logistic regression. Confounded variables present as an obstacle to valid inference in MetS studies. Hypertension and dyslipidemia are both common chronic conditions that affect a large proportion of the general adult population. Previous studies determining the association of FI and MetS also adjusted MetS-DCs[19]. Results of the adjusted model provide valid inference among MetS and insulin levels. A ROC curve was created for FI as a biomarker of MetS. The area under the ROC curve (AUC) was analyzed, and the optimized cut-off point for FI, sensitivity, and specificity were acquired

For peer review only

For peer review only - http://bmjopen.bmj.com/site/about/guidelines.xhtml

using the maximal Youden's Index. We used SPSS (version 23.0 for windows), to perform the statistical analysis. Statistical significance was set at a p-value < 0.05.

RESULTS

A total of 321 individuals, 111 men (34.6%) and 210 (65.4%) women, with a mean age of 63.91±8.32 years, were enrolled in this study. There were 90 study participants (28%) who met the diagnosis of MetS.

Table 1 shows the characteristics of the study population, which was divided based on the FI level in μU/mL. There were no statistically significant differences in age or gender between the low, middle, and high insulin level groups, while differences did exist with respect to WC, SBP, HDL-C, TG, and the proportion with MetS. Table 2 further shows the correlation between the FI level and all MetS-DCs, even after adjusting for age. FI was positively correlated with SBP, WC, FPG, and TG, and negatively correlated with HDL-C, as shown in Table 2. Table 3 shows the prevalence of MetS-DCs (hypertension, hyperglycemia, dyslipidemia, and central obesity) according to the insulin level tertiles. The prevalence of MetS-DCs increased as the FI level increased, as shown by significant p values (Cochran-Armitage trend test). Figure 1 shows that the low insulin level group had a 10% prevalence of MetS, the middle insulin level group had a 21.5% prevalence of MetS, and the high insulin level group had a 53.8% of MetS (p<0.0001 [Cochran-Armitage trend test]), suggesting that the prevalence of MetS increased with an increase in FI levels.

When designating the low insulin level group as the reference, the middle and high insulin level groups had an OR of 2.46 (p=0.02) and 10.50 (p<0.001) for MetS, respectively. After adjusting age, gender, and BMI, the middle and high insulin level groups had an OR of 1.71 (p=0.20) and 5.63 (p<0.001) for MetS, respectively. After adjusting age, gender, BMI, smoking, hypertension and dyslipidemia, the middle and high insulin level groups still had an OR of 1.51 (p=0.35) and 5.04 (p<0.001; Table 4) for MetS, respectively. There was no

For peer review only - http://bmjopen.bmj.com/site/about/guidelines.xhtml

significant difference between the middle and low tertile groups, but a significant difference between the high and low tertile groups, even after adjusting for the above risk factors. Based on this data, the high insulin level group had a 5-fold risk for MetS compared to the low insulin level group.

Figure 2 shows the ROC curve of FI as a biomarker for MetS. The AUC was 0.78. The optimized cut-off value for insulin was 7.35 μU/mL, with a sensitivity of 0.69 and a specificity of 0.77.

DISCUSSION

In this community-based study, we investigated fasting serum insulin levels in association with the prevalence of MetS in non-diabetic middle-aged and elderly Taiwanese adults. In our study, the prevalence of MetS in the relatively healthy middle age-to-elderly population was 28%, which is similar to the 29.75% findings reported by Li et al.[24] Among middle-aged and elderly populations in Taiwan. Looking at the three FI tertiles, there was a rising proportion of MetS as the FI level increased, also shown in previous studies[20 25 26]. This finding not only applied to MetS, but to MetS-DCs as well. The WC, SBP, TG, and FPG levels were the lowest in the low FI group and highest in the high FI group; the converse applied to HDL and vice versa (Table 1). This finding led us to speculate that an association exists between FI levels and MetS-DCs. We found a statistically significant correlation between FI levels and each MetS-DC, even after adjusting for age (Table 2). A trend existed between the FI level and the prevalence of all five MetS-DCs (Table 3). We thus wanted to know if the trend applied to the prevalence of MetS. Figure 1 shows an apparent increase in the prevalence of MetS as the FI level increased. The trend was confirmed by the Cochran-Armitage trend test ($p<0.0001$).

After adjusting for gender, age, BMI, smoking status, hypertension, and dyslipidemia, the middle-aged and elderly populations in the high FI group were at significant risk for

For peer review only

For peer review only - http://bmjopen.bmj.com/site/about/guidelines.xhtml

developing MetS (OR = 5.04, 95% CI =2.15-11.81; P < 0.01: Table 4). This conclusion is consistent with previous findings[20 26 27]. Not only is FI an independent risk factor for MetS, but in the cohort study of Sung et al., it was also reported that elevated FI predicted the future incidence of MetS[19]. One possible explanation might be the relationship between the FI level and insulin resistance[20 21], which has a fundamental role in MetS[28 29]. Although the mechanism by which FI may represent insulin resistance was not investigated in the present study, a number of studies have shown that FI is a suitable surrogate marker for insulin resistance[21 30-33], calculated by the fasting insulin resistance index (FIRI) or homeostasis model assessment of insulin resistance (HOMA-IR). A higher FI level is associated with insulin resistance in patients with impaired fasting glucose, but may be an inappropriate marker in diabetics with poor glycemic control. It has been reported that the FI level is highly associated with MetS[20]. In our study, the AUC for FI as an indicator for MetS was 0.78, similar to another study's AUC of 0.77[20]. Based on our search of the literature, there is no widely accepted reference range for FI. A reference range for FI of 1.57–16.32 μU/mL has been proposed in Chinese men, but the reference range varies between different ethnicities and genders[34]. A FI level above 9μU/mL has been reported to identify 80% of patients with pre-diabetes[35]. Although we obtained a cut-off value for fasting insulin, but due to large variations in insulin assays, this value of >7.35 should not be generalized to other laboratory sites.

Our findings may have an impact on health screening policies in non-diabetic people older than middle-age. Elevated FI may act as an accompanying marker to enhance the risk of MetS. We do not propose to discard MetS criteria, but suggest that elevated FI may alert physicians on the risk of MetS in clinical settings of non-diabetic individuals. Given the fact that elevated FI is not only associated with a greater risk for developing MetS[19 36] but is also associated with a greater number of cardiometabolic risk factors[22], healthy behavior should

For peer review only - http://bmjopen.bmj.com/site/about/guidelines.xhtml

be considered when the FI level is relatively higher in the population. Though we are in need of large trials to determine if subjects with early stages of insulin resistance can benefit from interventions.

Some limitations in our study merit consideration. First, the principal limitation relevant to the interpretation of our results was the use of a cross-sectional design, thus a causal relationship between the FI level and MetS cannot be inferred. Second, the sample size in our study was relatively small (n=321[the power was not calculated]) and the participants were recruited from a regional community. The participants could therefore only be distributed into three groups and the results cannot be generalized to other ethnicities. Third, though males tend to have a lower participation rate in studies[37], there may still have been a selection bias due to the higher participation of women than men in our study. Fourth, the FI cutoff value varies between different ethnic groups and insulin assays, so physicians should be aware of this variation in clinical settings. Besides, the false negative rate (31%) should be taken into consideration when applying this data. Furthermore, even though we used a standardized questionnaire, recall and reporting bias are unavoidable for self-reported data. Finally, we did not ask participants to sleep adequately or to avoid vigorous exercise the day before blood testing, which could affect the accuracy of the fasting serum insulin level.

Our study also has strengths. First, our participants were recruited during a community health examination and represent a relatively healthy population. The effects of important confounders, including ethnicity, residential area, and environmental factors, were minimized. Second, we used standardized laboratory examination protocols and anthropometric measurements. Third, while evaluating the association between MetS and the FI level, we excluded diabetic patients to avoid the effect of anti-diabetic medications on the FI level. Lastly, due to the trend of world aging, our study aimed for middle-aged and elderly populations. Studies from all around the world indicate the relationship of FI and MetS (table

For peer review only

For peer review only - http://bmjopen.bmj.com/site/about/guidelines.xhtml

5), our study contributes to the Taiwanese population.

In the future, we will continue to follow this community and record the development of newly diagnosed MetS. Counseling of healthy behaviors for residents with elevated FI will also be our topic of interest hereon. Whether lifestyle modification could retard the development of MetS in high FI individuals requires further studies to elaborate.

CONCLUSIONS

Our study provides a method to identify the risk of MetS by testing FI levels in the middle aged and elderly non-diabetic populations. When a non-diabetic individual is presented with a high FI level, physicians may be alerted of the risk of MetS. Our study confirms the association between FI and MetS. Further prospective research is needed to clarify the link between FI and MetS.

Contributors YHC and YCL were involved in writing of the manuscript. YCT, MCL, HHC and WCY conceived and supervised the study. IST provided statistical advice. JYC contributed conceived, designed and performed the experiments, collected and analyzed the data, revising it critically for important intellectual content and final approval of the version to be submitted.

Funding This work was supported by Chang Gung Memorial Hospital (CORPG3C0171, CORPG3C0172, CZRPG3C0053).

Competing interests None declared.

Ethics approval The study was approved by Chang-Gung Medical Foundation Institutional Review Board (102-2304B), and written informed consent was given by all the participants before enrollment.

Data sharing statement No additional data are available.

For peer review only

REFERENCES

1. Kaur J. A comprehensive review on metabolic syndrome. *Cardiol Res Pract* 2014;2014:943162. doi: 10.1155/2014/943162

2. Nakao YM, Miyawaki T, Yasuno S, et al. Intra-abdominal fat area is a predictor for new onset of individual components of metabolic syndrome: MEtabolic syndRome and abdominaL ObesiTy (MERLOT study). *Proc Jpn Acad Ser B Phys Biol Sci* 2012;88(8):454-61.

3. Popa S, Mota M, Popa A, et al. Prevalence of overweight/obesity, abdominal obesity and metabolic syndrome and atypical cardiometabolic phenotypes in the adult Romanian population: PREDATORR study. *J Endocrinol Invest* 2016;39(9):1045-53. doi: 10.1007/s40618-016-0470-4

4. Li W-C, Chen J-Y, Lin C-H, et al. Reevaluating the Diagnostic Criteria for Metabolic Syndrome in the Taiwanese Population. *Journal of the American College of Nutrition* 2011;30(4):241-47.

5. Dragsbaek K, Neergaard JS, Laursen JM, et al. Metabolic syndrome and subsequent risk of type 2 diabetes and cardiovascular disease in elderly women: Challenging the current definition. *Medicine (Baltimore)* 2016;95(36):e4806. doi: 10.1097/MD.0000000000004806

6. Meifang Y, Xue S, Jue H, et al. [Metabolic syndrome increases Framingham risk score of patients with type 2 diabetes mellitus]. *Zhejiang Da Xue Xue Bao Yi Xue Ban* 2016;45(3):268-74.

7. Oguoma VM, Nwose EU, Skinner TC, et al. Association between metabolic syndrome and 10-year risk of developing cardiovascular disease in a Nigerian population. *Int Health* 2016 doi: 10.1093/inthealth/ihw013

8. Desroches S, Lamarche B. The evolving definitions and increasing prevalence of the

For peer review only

For peer review only - http://bmjopen.bmj.com/site/about/guidelines.xhtml

metabolic syndrome. *Appl Physiol Nutr Metab* 2007;32(1):23-32. doi: 10.1139/h06-095
9. Ford ES, Giles WH, Mokdad AH. Increasing prevalence of the metabolic syndrome among u.s. Adults. *Diabetes Care* 2004;27(10):2444-9.
10. Hu G, Lindstrom J, Jousilahti P, et al. The increasing prevalence of metabolic syndrome among Finnish men and women over a decade. *J Clin Endocrinol Metab* 2008;93(3):832-6. doi: 10.1210/jc.2007-1883
11. Lim S, Shin H, Song JH, et al. Increasing prevalence of metabolic syndrome in Korea: the Korean National Health and Nutrition Examination Survey for 1998-2007. *Diabetes Care* 2011;34(6):1323-8. doi: 10.2337/dc10-2109
12. Liu M, Wang J, Jiang B, et al. Increasing Prevalence of Metabolic Syndrome in a Chinese Elderly Population: 2001-2010. *PLoS One* 2013;8(6):e66233. doi: 10.1371/journal.pone.0066233
13. Grundy SM, Brewer HB, Jr., Cleeman JI, et al. Definition of metabolic syndrome: Report of the National Heart, Lung, and Blood Institute/American Heart Association conference on scientific issues related to definition. *Circulation* 2004;109(3):433-8. doi: 10.1161/01.CIR.0000111245.75752.C6
14. Lann D, LeRoith D. Insulin resistance as the underlying cause for the metabolic syndrome. *Med Clin North Am* 2007;91(6):1063-77, viii. doi: 10.1016/j.mcna.2007.06.012
15. Gallagher EJ, Leroith D, Karnieli E. Insulin resistance in obesity as the underlying cause for the metabolic syndrome. *Mt Sinai J Med* 2010;77(5):511-23. doi: 10.1002/msj.20212
16. Dontsov AV, Vasil'eva LV. [Insulin Resistance Associated with Metabolic Syndrome as an Indicator of Cardiovascular Risk]. *Klin Med (Mosk)* 2016;94(3):189-93.
17. Polonsky KS, Given BD, Van Cauter E. Twenty-four-hour profiles and pulsatile patterns of insulin secretion in normal and obese subjects. *J Clin Invest* 1988;81(2):442-8. doi: 10.1172/JCI113339

For peer review only - http://bmjopen.bmj.com/site/about/guidelines.xhtml

18. Wilcox G. Insulin and insulin resistance. *Clin Biochem Rev* 2005;26(2):19-39.

19. Sung KC, Seo MH, Rhee EJ, et al. Elevated fasting insulin predicts the future incidence of metabolic syndrome: a 5-year follow-up study. *Cardiovasc Diabetol* 2011;10:108. doi: 10.1186/1475-2840-10-108

20. Saravia G, Civeira F, Hurtado-Roca Y, et al. Glycated Hemoglobin, Fasting Insulin and the Metabolic Syndrome in Males. Cross-Sectional Analyses of the Aragon Workers' Health Study Baseline. *PLoS One* 2015;10(8):e0132244. doi: 10.1371/journal.pone.0132244

21. ter Horst KW, Gilijamse PW, Koopman KE, et al. Insulin resistance in obesity can be reliably identified from fasting plasma insulin. *Int J Obes (Lond)* 2015;39(12):1703-9. doi: 10.1038/ijo.2015.125 [published Online First: 2015/07/15]

22. Pataky Z, Golay A, Laville M, et al. Fasting insulin at baseline influences the number of cardiometabolic risk factors and R-R interval at 3years in a healthy population: the RISC Study. *Diabetes Metab* 2013;39(4):330-6. doi: 10.1016/j.diabet.2013.05.008 [published Online First: 2013/07/24]

23. Tan CE, Ma S, Wai D, et al. Can we apply the National Cholesterol Education Program Adult Treatment Panel definition of the metabolic syndrome to Asians? *Diabetes Care* 2004;27(5):1182-6.

24. Li Z, Yang X, Yang J, et al. The Cohort Study on Prediction of Incidence of All-Cause Mortality by Metabolic Syndrome. *PLoS One* 2016;11(5):e0154990. doi: 10.1371/journal.pone.0154990

25. Kanda H, Wang P, Okamura T, et al. Fasting plasma insulin is associated with metabolic syndrome in farmers but not in nomads among the Mongolian population, China. *J Atheroscler Thromb* 2011;18(4):291-7.

26. Prevalence of the metabolic syndrome among a racially/ethnically diverse group of U.S.

For peer review only - http://bmjopen.bmj.com/site/about/guidelines.xhtml

eighth-grade adolescents and associations with fasting insulin and homeostasis model assessment of insulin resistance levels. *Diabetes Care* 2008;31(10):2020-5. doi: 10.2337/dc08-0411 [published Online First: 2008/07/02]

27. Adam FM, Nara MG, Adam JM. Fasting insulin, adiponectin, hs-CRP levels, and the components of metabolic syndrome. *Acta Med Indones* 2006;38(4):179-84.
28. Saltiel AR. Series introduction: the molecular and physiological basis of insulin resistance: emerging implications for metabolic and cardiovascular diseases. *J Clin Invest* 2000;106(2):163-4. doi: 10.1172/JCI10533
29. Mayans L. Metabolic Syndrome: Insulin Resistance and Prediabetes. *FP Essent* 2015;435:11-6.
30. Duncan MH, Singh BM, Wise PH, et al. A simple measure of insulin resistance. *Lancet* 1995;346(8967):120-1.
31. Abbasi F, Reaven GM. Evaluation of the quantitative insulin sensitivity check index as an estimate of insulin sensitivity in humans. *Metabolism* 2002;51(2):235-7.
32. Kim SH, Abbasi F, Reaven GM. Impact of degree of obesity on surrogate estimates of insulin resistance. *Diabetes Care* 2004;27(8):1998-2002.
33. Tang Q, Li X, Song P, et al. Optimal cut-off values for the homeostasis model assessment of insulin resistance (HOMA-IR) and pre-diabetes screening: Developments in research and prospects for the future. *Drug Discov Ther* 2015;9(6):380-5. doi: 10.5582/ddt.2015.01207
34. Li S, Huang S, Mo ZN, et al. Generating a reference interval for fasting serum insulin in healthy nondiabetic adult Chinese men. *Singapore Med J* 2012;53(12):821-5.
35. Johnson JL, Duick DS, Chui MA, et al. Identifying prediabetes using fasting insulin levels. *Endocr Pract* 2010;16(1):47-52. doi: 10.4158/EP09031.OR
36. Rutter MK, Sullivan LM, Fox CS, et al. Baseline levels, and changes over time in body

For peer review only

For peer review only - http://bmjopen.bmj.com/site/about/guidelines.xhtml

mass index and fasting insulin, and their relationship to change in metabolic trait clustering. *Metab Syndr Relat Disord* 2014;12(7):372-80. doi: 10.1089/met.2013.0148

37. Galea S, Tracy M. Participation rates in epidemiologic studies. *Ann Epidemiol* 2007;17(9):643-53. doi: 10.1016/j.annepidem.2007.03.013 [published Online First: 2007/06/08]

Figure Legends

Figure 1. Prevalence of metabolic syndrome based on insulin levels. A linear increasing trend across insulin tertiles.

Figure 2. ROC curve for insulin as a biomarker of metabolic syndrome. AUC 0.78. FI 7.35μU/mL (sensitivity: 0.69; specificity: 0.77).

For peer review only - http://bmjopen.bmj.com/site/about/guidelines.xhtml

Table 1 General characteristics of the study population based on insulin levels.

	Insulin levels				
	**Total**	**Low**	**Middle**	**High**	
**Variables**	**n=321**	**n=110 (≦4.8)**	**n=107 (4.9-7.8)**	**n=104 (≧7.9)**	**p value**
Age (year)	63.91 ±8.32	64.23 ±8.32	64.47 ±8.67	63.01 ±7.93	0.40
BMI (kg/m^2)	24.36 ±3.53	22.41 ±3.14	24.41 ±2.73	26.37 ±3.54	<0.001
Waist circumference (cm)	84.23 ±9.51	79.69 ±7.57	83.78 ±8.63	89.51 ±9.65	<0.001
SBP (mmHg)	129.02 ±16.59	123.69 ±17.36	129.52 ±14.51	134.13 ±16.20	<0.001
DBP (mmHg)	77.01 ±10.90	75.43 ±11.80	76.92 ±10.03	78.79 ±10.60	0.08
ALT (U/L)	21.74 ±11.06	18.94 ±7.81	20.33 ±9.25	26.15 ±14.05	<0.001
Creatinine (mg/dL)	0.76 ±0.44	0.69 ±0.17	0.85 ±0.66	0.75 ±0.34	0.03
FPG (mg/dL)	89.10 ±9.93	85.29 ±9.11	89.18 ±8.52	93.05 ±10.60	<0.001
HDL-C (mg/dL)	55.70 ±14.05	60.93 ±14.85	55.59 ±13.17	50.28 ±11.94	<0.001
Insulin (μU/mL)	7.10 ±4.14	3.60 ±0.94	6.21 ±0.86	11.72 ±4.02	<0.001
LDL-C (mg/dL)	121.48 ±32.05	118.90 ±34.55	126.03 ±31.01	119.53 ±30.10	0.20
T-cholesterol (mg/dL)	200.61 ±35.20	198.85 ±36.98	203.81 ±35.12	119.18 ±33.43	0.52
TG (mg/dL)	117.34 ±60.61	95.39 ±45.13	111.04 ±49.83	147.05 ±72.48	<0.001
Current smoking, n(%)	34 (10.6)	14 (12.7)	11 (10.3)	9 (8.7)	0.62
Marital status (single), n(%)	54 (16.8)	22 (20.0)	14 (13.1)	18 (17.3)	0.39
Men, n(%)	111 (34.6)	41 (37.3)	39 (36.4)	31 (29.8)	0.46
Regular exercise, n(%)	264 (82.2)	92 (83.6)	96 (89.7)	76 (73.1)	0.01
Vegetarian, n(%)	20 (6.2)	7 (6.4)	7 (6.5)	6 (5.8)	0.97
HTN, n(%)	150 (46.7)	43 (39.1)	47 (43.9)	60 (57.7)	0.02
Hyperlipidemia, n(%)	204 (63.6)	58 (52.7)	69 (64.5)	77 (74.0)	0.005
Metabolic syndrome, n(%)	90 (28.0)	11 (10.0)	23 (21.5)	56 (53.8)	<0.001

Notes: Ranges of FI levels of different tertile groups are shown in brackets at the top of the table, units in μU/mL.

Clinical characteristics are expressed as the mean±SD for continuous variables and n(%) for categorical variables. P-values were derived from one-way analysis of variance (ANOVA) for

For peer review only

For peer review only - http://bmjopen.bmj.com/site/about/guidelines.xhtml

continuous variables and chi-square test for categorical variables.

Abbreviations: BMI, body mass index; SBP, systolic blood pressure; DBP, diastolic blood pressure; ALT, alanine aminotransferase; FPG, fasting plasma glucose; HDL-C, high-density lipoprotein cholesterol; LDL-C, low-density lipoprotein cholesterol; TG, triglyceride; HTN, hypertension.

Table 2 Pearson's correlation coefficients for each component of metabolic syndrome and age in relation to insulin levels.

Variables	Insulin(n=321)			
	Unadjusted		Adjusted for age	
	Pearson's coefficient	p value	Pearson's coefficient	p value
Age (year)	-0.04	0.50	NA	NA
SBP (mmHg)	0.21	<0.001	0.22	<0.001
DBP (mmHg)	0.11	0.05	0.10	0.07
Waist circumference (cm)	0.43	<0.001	0.44	<0.001
FPG (mg/dL)	0.38	<0.001	0.39	<0.001
HDL-C (mg/dL)	-0.37	<0.001	-0.37	<0.001
TG (mg/dL)	0.37	<0.001	0.37	<0.001

Abbreviations: SBP, systolic blood pressure; DBP, diastolic blood pressure; FPG, fasting plasma glucose; HDL-C, high-density lipoprotein cholesterol; TG, triglyceride.

For peer review only - http://bmjopen.bmj.com/site/about/guidelines.xhtml

Table 3 Prevalence of components of metabolic syndrome based on insulin levels.

Components	Low (n=110) N(%)	Middle (n=107) N(%)	High (n=104) N(%)	p value for Cochran-Armitage trend test
High blood pressure[a]	56(50.9)	63(58.9)	78(75)	0.0003
High blood glucose[b]	8(7.3)	10(9.3)	25(24.0)	0.0004
Low HDL-C[c]	15(13.6)	19(17.8)	43(41.3)	<0.0001
High TG[d]	17(15.5)	27(25.2)	42(40.4)	<0.0001
Central obesity[e]	34(30.9)	57(53.3)	82(78.8)	<0.0001

Note:

[a] SBP≧130 mmHg or DBP≧85 mmHg, or self-reported hypertension

[b] Fasting blood glucose ≥100 mg/dL or self-reported diabetes mellitus

[c] HDL-C<40 mg/dL in men or <50 mg/dL in women

[d] TG≥150 mg/dL

[e] Waist circumference ≥90 cm in men or ≥80 cm in women

Abbreviations: HDL-C, high-density lipoprotein-cholesterol; TG, triglyceride; SBP, systolic blood pressure; DBP, diastolic blood pressure.

For peer review only

Table 4 Association between insulin levels and metabolic syndrome.

Variables	Model 1			Model 2			Model 3		
	**OR**	**(95%CI)**	**p value**	**OR**	**(95%CI)**	**p value**	**OR**	**(95%CI)**	**p value**
Low	1.00	—	—	1.00	—	—	1.00	—	—
Middle	2.46	(1.14-5.35)	0.02	1.71	(0.76-3.85)	0.20	1.51	(0.64-3.57)	0.35
High	10.50	(5.05-21.84)	<0.001	5.63	(2.53-12.53)	<0.001	5.04	(2.15-11.81)	<0.001
p value for trend			<0.001			<0.001			<0.001

Model 1: unadjusted

Model 2: adjusted for gender, age, and BMI

Model 3: adjusted for factors in model 2 plus smoking, HTN, and hyperlipidemia

Abbreviations: BMI, body mass index; HTN, hypertension; OR, odds ratio; CI, confidence interval.

For peer review only

For peer review only - http://bmjopen.bmj.com/site/about/guidelines.xhtml

Table 5 Studies of Association Between Fasting Insulin and Metabolic Syndrome.

Authors	Study Year	Study Population	% MetS	Fasting insulin	Risk of MetS	Main Finding	Reference
Saravia G et al 2015	2009-2010 Cross sectional	3200 Non-diabetic males in Spain	23%	Highest tertile (≥6.13) vs lowest (≤3.80)µU/mL	OR (95%CI) 11.36 (8.65-15.13) for MetS	Per each 10 pmol/L (1.4 uU/mL) increase in insulin, the odds for metabolic syndrome increased by 1.43 (95%CI: 1.38, 1.49)	[20]
Rutter MK et al 2014	(1991-1995) to (1998-2001) 7 year Prospective	2616 non-diabetic adults in Europe	-	1-quintile change in fasting insulin (pmol/L)	mean (95% CI) 0.12 (0.10–0.15) [MetS trait score 7 year change]	Change in metabolic trait clustering was significantly associated with baseline levels and changes in fasting insulin.	[38]
Sung KC et al 2011	2003-2008 5 year cohort	2350 non-MetS in Korea	8.5% (incidence)	Highest quartile (≥8.98) vs lowest (≤6.01) IU/ml	OR (95% CI) of developing MS 5.1 (3.1-8.2)	The highest quartile of the insulin levels had more than a 5 times greater risk of developing MS compared to the subjects in the lowest quartile.	[19]
Kanda H et al 2011	2000,2001 Cross sectional	456 in Mongolia	6.4%	Highest tertile (≥10.33) vs lowest (≤6.72) mmol/L	Percentage of MetS 17.1% vs 4.6%	Fasting plasma insulin is associated with MetS in farmers, but not nomads among the Mongolian population in China.	[27]
STOPP-T2D PSG* 2008	2003 Cross sectional	1453 8th grade adolescents in the U.S.	9.5%	Highest quintile (≥39.1) vs lowest (≤17.0) µU/mL	OR (95%CI) 199.64(31.29-1273.7) for MetS	The highest insulin quintile were almost 200 times more likely to be classified with the metabolic syndrome than participants in the lowest quintile.	[28]
Adam FM et al 2006	2005 Cross sectional	128 overweight/ obese in Indonesia	68.8%	Mean fasting insulin levels15.68±7.85 vs 3.16±2.53 (uU/ml) with 5 components vs 1 component of MetS.		There is a strong linear increase in fasting insulin levels with an increase of the number of metabolic syndrome.	[29]

*STOPP-T2D PSG: Studies to Treat or Prevent Pediatric Type 2 Diabetes Prevention Study Group

For peer review only - http://bmjopen.bmj.com/site/about/guidelines.xhtml

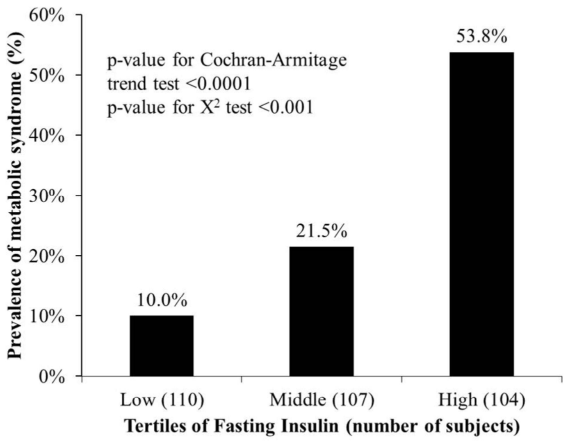

Figure 1 Prevalence of metabolic syndrome based on insulin levels. A linear increasing trend across insulin tertiles.

Figure 1. Prevalence of metabolic syndrome based on insulin levels. A linear increasing trend across insulin tertiles.

297x209mm (300 x 300 DPI)

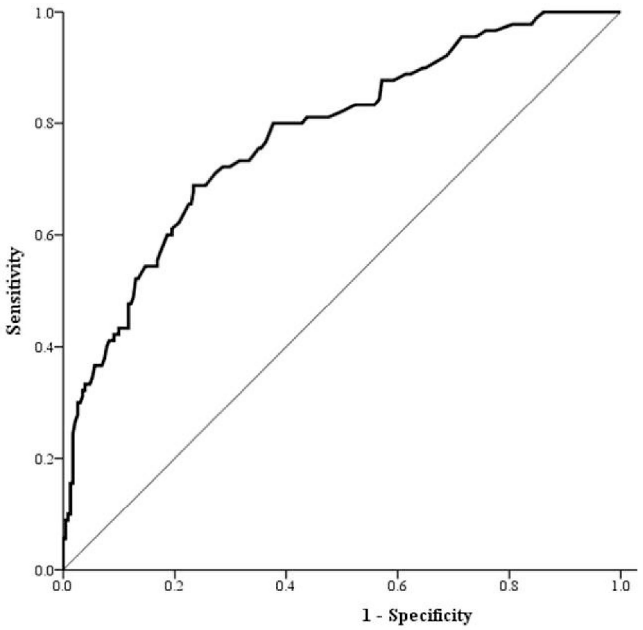

Figure 2. ROC curve for insulin as a biomarker of metabolic syndrome. AUC 0.78. FI 7.35μU/mL (sensitivity: 0.69; specificity: 0.77).

Figure 2. ROC curve for insulin as a biomarker of metabolic syndrome. AUC 0.78. FI 7.35μU/mL (sensitivity: 0.69; specificity: 0.77).

297x209mm (300 x 300 DPI)

STROBE 2007 (v4) Statement—Checklist of items that should be included in reports of *cross-sectional studies*

Section/Topic	Item #	Recommendation	Reported on page #
**Title and abstract**	1	(*a*) Indicate the study's design with a commonly used term in the title or the abstract	P2
		(*b*) Provide in the abstract an informative and balanced summary of what was done and what was found	P2
**Introduction**			
Background/rationale	2	Explain the scientific background and rationale for the investigation being reported	P4
Objectives	3	State specific objectives, including any prespecified hypotheses	P2, P4
**Methods**			
Study design	4	Present key elements of study design early in the paper	P4
Setting	5	Describe the setting, locations, and relevant dates, including periods of recruitment, exposure, follow-up, and data collection	P4, 5
Participants	6	(*a*) Give the eligibility criteria, and the sources and methods of selection of participants	P4
Variables	7	Clearly define all outcomes, exposures, predictors, potential confounders, and effect modifiers. Give diagnostic criteria, if applicable	P5
Data sources/ measurement	8*	For each variable of interest, give sources of data and details of methods of assessment (measurement). Describe comparability of assessment methods if there is more than one group	P5
Bias	9	Describe any efforts to address potential sources of bias	Nil
Study size	10	Explain how the study size was arrived at	Nil
Quantitative variables	11	Explain how quantitative variables were handled in the analyses. If applicable, describe which groupings were chosen and why	
Statistical methods	12	(*a*) Describe all statistical methods, including those used to control for confounding	P6
		(*b*) Describe any methods used to examine subgroups and interactions	P6
		(*c*) Explain how missing data were addressed	Nil
		(*d*) If applicable, describe analytical methods taking account of sampling strategy	Nil
		(*e*) Describe any sensitivity analyses	Nil
**Results**			

For peer review only

Participants	13*	(a) Report numbers of individuals at each stage of study—eg numbers potentially eligible, examined for eligibility, confirmed eligible, included in the study, completing follow-up, and analysed	Nil
		(b) Give reasons for non-participation at each stage	Nil
		(c) Consider use of a flow diagram	Nil
Descriptive data	14*	(a) Give characteristics of study participants (eg demographic, clinical, social) and information on exposures and potential confounders	P8
		(b) Indicate number of participants with missing data for each variable of interest	Nil
Outcome data	15*	Report numbers of outcome events or summary measures	P6-9
Main results	16	(*a*) Give unadjusted estimates and, if applicable, confounder-adjusted estimates and their precision (eg, 95% confidence interval). Make clear which confounders were adjusted for and why they were included	P7
		(*b*) Report category boundaries when continuous variables were categorized	P8
		(*c*) If relevant, consider translating estimates of relative risk into absolute risk for a meaningful time period	Nil
Other analyses	17	Report other analyses done—eg analyses of subgroups and interactions, and sensitivity analyses	Nil
**Discussion**			
Key results	18	Summarise key results with reference to study objectives	P7
Limitations	19	Discuss limitations of the study, taking into account sources of potential bias or imprecision. Discuss both direction and magnitude of any potential bias	P17
Interpretation	20	Give a cautious overall interpretation of results considering objectives, limitations, multiplicity of analyses, results from similar studies, and other relevant evidence	P17
Generalisability	21	Discuss the generalisability (external validity) of the study results	P18
**Other information**			
Funding	22	Give the source of funding and the role of the funders for the present study and, if applicable, for the original study on which the present article is based	P19

*Give information separately for cases and controls in case-control studies and, if applicable, for exposed and unexposed groups in cohort and cross-sectional studies.

Note: An Explanation and Elaboration article discusses each checklist item and gives methodological background and published examples of transparent reporting. The STROBE checklist is best used in conjunction with this article (freely available on the Web sites of PLoS Medicine at http://www.plosmedicine.org/, Annals of Internal Medicine at http://www.annals.org/, and Epidemiology at http://www.epidem.com/). Information on the STROBE Initiative is available at www.strobe-statement.org.

For peer review only

BMJ Open

Association between high fasting insulin levels and metabolic syndrome in non-diabetic middle-aged and elderly populations: A community-based study in Taiwan

Journal:	*BMJ Open*
Manuscript ID	bmjopen-2017-016554.R3
Article Type:	Research
Date Submitted by the Author:	03-Nov-2017
Complete List of Authors:	Chen, Yun-Hung; Chang Gung Memorial Hospital Linkou Branch, Family Medicine<br>Lee, Yu-Chien; Chang Gung Memorial Hospital Linkou Branch, Family Medicine<br>Tsao, Yu-Chung; Chang Gung Memorial Hospital Linkou Branch, Family Medicine; Chang Gung Memorial Hospital Linkou Branch, Occupational Medicine<br>Lu, Mei-Chun; Chang Gung Memorial Hospital Linkou Branch, Family Medicine<br>Chuang, Hai-Hua; Chang Gung Memorial Hospital Taipei Branch, Family Medicine<br>Yeh, Wei-Chung; Chang Gung Memorial Hospital Linkou Branch, Family Medicine; Chang Gung University College of Medicine<br>Tzeng, I-Shiang; Taipei Tzu Chi General Hospital, Department of Research<br>Chen, Jau-Yuan; Chang Gung Memorial Hospital Linkou Branch, Family Medicine; Chang Gung University College of Medicine
**Primary Subject Heading**:	General practice / Family practice
Secondary Subject Heading:	General practice / Family practice, Geriatric medicine, Health policy
Keywords:	metabolic syndrome, insulin resistance, fasting insulin level

SCHOLARONE™
Manuscripts

- **Title**

Association between high fasting insulin levels and metabolic syndrome in non-diabetic middle-aged and elderly populations: A community-based study in Taiwan

- **Short title**

Increased fasting insulin levels in association with metabolic syndrome

- **Author names and affiliations**

Yun-Hung Chen[1], Yu-Chien Lee[1], Yu-Chung Tsao[1, 2, 3], Mei-Chun Lu[1], Hai-Hua Chuang[4], Wei-ChungYeh[1, 3], I-Shiang Tzeng[5], Jau-Yuan Chen[1, 3, *]

[1]Department of Family Medicine, Chang Gung Memorial Hospital, Linkou Branch, Taiwan

[2]Department of Occupational Medicine, Chang-Gung Memorial Hospital, Linkou Branch, Taiwan

[3]College of Medicine, Chang Gung University, Taoyuan, Taiwan

[4]Department of Family Medicine, Chang-Gung Memorial Hospital, Taipei Branch, Taiwan

[5]Department of Research, Taipei Tzu Chi Hospital, Buddhist Tzu Chi Medical Foundation, New Taipei city, Taiwan

*Corresponding author

E-mail: welins@cgmh.org.tw (J.-Y. Chen)

Postal address: No. 5, Fuxing St., Guishan Dist., Taoyuan City 333, Taiwan (R.O.C.)

Phone: +886-975362672

Fax: +886-3-3287715

- **Format**

Word count: 2491

Number of black and white figures: 2

Number of Tables: 5

Number of References: 37

For peer review only

ABSTRACT

Objectives: We aimed to determine the association between fasting insulin (FI) levels and metabolic syndrome (MetS) in non-diabetic middle-aged and elderly adults in a community in Taiwan.

Design: Cross-sectional observational study.

Setting: Community-based investigation in Guishan township of northern Taiwan.

Participants: Our study included adults aged 50 years and above during community health exams between January and October 2014. People with diabetes mellitus were excluded. A total of 321 people were enrolled.

Outcome measures: We divided participants according to tertiles of FI as low, medium, and high levels. Pearson correlation was assessed between insulin level and each of the diagnostic components of metabolic syndrome (MetS-DCs) with adjustment of age. The prevalence of MetS-DCs based on tertiles of FI were studied and analyzed by Cochran-Armitage trend test. The risk for prevalence of MetS in the middle, and high insulin group as compared to the low insulin group were assessed by multivariate logistic regression with adjustments for age, gender, smoking, body mass index (BMI), hypertension, and hyperlipidemia. Youden Index was performed for the optimized cut-off value.

Results: Our results showed positive correlation of FI level with systolic blood pressure, waist circumference, fasting plasma glucose and triglyceride levels, while negative correlation was shown with high-density lipoprotein ($p<0.001$). The prevalence of each MetS-DCs increased as a trend while FI levels increased ($p<0.001$). OR (95% CI) of MetS was 5.04 (2.15-11.81) for high insulin groups compared to the low insulin group after adjusting confounders ($p<0.001$). Area under receiver operating characteristic curve (ROC) curve (AUC) was 0.78, and cut-off value 7.35 μU/mL for FI was obtained (sensitivity: 0.69; specificity: 0.77).

For peer review only

Conclusions: Middle-aged and elderly non-diabetic people with increased FI are associated with a higher prevalence of MetS in the community in Taiwan. Furthermore, FI is an independent risk factor of MetS in this study population.

Keywords: metabolic syndrome, insulin resistance, fasting insulin level

Strengths and limitations of this study:

- This is the first study to explore the relationship between fasting insulin and MetS in the middle-aged and elderly populations of non-diabetic Asian people.
- We offer a biomarker to identify middle-aged and elder non-diabetic Taiwanese with MetS.
- We adjusted many confounding factors to make the results more reliable.
- The recall bias from self-reported lifestyle behaviors is unavoidable.
- The causal relationship between fasting insulin level and MetS cannot be demonstrated in our cross-sectional study.

For peer review only

For peer review only - http://bmjopen.bmj.com/site/about/guidelines.xhtml

INTRODUCTION

Metabolic syndrome (MetS) is associated with a cluster of unhealthy metabolic risk factors, including abdominal obesity (excess body fat around the waist), glucose intolerance, pre-morbid hypertension, and dyslipidemia[1-3]. A number of studies have reported that MetS increases the risk of type 2 diabetes mellitus (T2DM), cardiovascular disease (CVD) and other non-communicable diseases (NCDs)[4-7]. The rising prevalence of MetS has created a disturbing challenge to personal health[8-12].

Insulin resistance has long been associated with MetS[13-16]. Basal insulin represents 45%-50% of daily insulin[17 18], and the FI level approximates basal insulin[18 19]. Studies have shown that FI levels are associated with the prevalence of MetS, which may be due to its representativeness of insulin resistance[20 21]. A study has even shown that elevated FI levels may predict the future incidence of MetS[19]. If insulin resistance is the foundation of MetS[14 15], and FI represents insulin resistance with an area under curve (AUC) [95% confidence interval (CI)] of 0.995[0.993-0.996] [20], a high FI level may be able to caution the physician for susceptibility to metabolic diseases and hence cardiovascular risks[22]. We therefore aimed to determine the association between FI levels and MetS in non-diabetic middle-aged and elderly adults in a community in Taiwan.

METHODS

Study subjects

This was an observational and cross-sectional study conducted at Linkou Chang Gung Memorial Hospital in Taoyuan County, Taiwan between January and October 2014. The inclusion criteria included residents 50-90 years old and living in Guishan township. 619 residents were eligible for the study. A total of 400 residents agreed to participate in our health exam. Subjects were excluded if they had diabetes. 79 participants with diabetes

For peer review only - http://bmjopen.bmj.com/site/about/guidelines.xhtml

mellitus were excluded. Diabetes mellitus was defined as any of the followings: 1. Previous diagnosis of diabetes mellitus; 2. Recent use of oral anti-hyperglycemic drugs or insulin; 3. Participants with fasting glucose≥126 mg/dl. A total of 321 participants (111 males and 210 females) were ultimately enrolled for analysis. This study was approved by the Institutional Review Board of the hospital and written informed consent was obtained from all of the participants before enrollment.

Data collection

We obtained exercise (exercising ≥3 times a week or not) and dietary habits (vegetarian or not) from self-administered questionnaires, which also included smoking (current smoker or not) and marital status (currently married or not). Anthropometric data, such as height, weight, waist circumference (WC), and blood pressure were also recorded. The subjects were dressed in light clothing without shoes for weight and height measurements. The BMI was calculated as the weight in kilograms (kg) divided by the height in meters squared (m^2). Waist circumference was measured midway between the inferior margin of the lowest rib and the iliac crest in the horizontal plane while in an upright position. Systolic blood pressure (SBP) and diastolic blood pressure (DBP) were checked at least 2 times after 5 min of rest while seated. FI levels, lipid profile, and fasting glucose were obtained by blood sampling after a 10-h overnight fast. Blood samples were analyzed in the central laboratory of Linkou Chang Gung Memorial Hospital for fasting plasma glucose (FPG), serum total cholesterol (TC), low-density lipoprotein-cholesterol (LDL-C), high-density lipoprotein-cholesterol (HDL-C), serum triglycerides (TG), and FI levels. Serum insulin levels were determined with an ARCHITECT Insulin assay (Abbott Laboratories, IL, USA). Insulin was measured with a chemiluminescent microparticle immunessay (CMIA). The intra-assay variation and inter-assay variations were less than 2.7%. The ARCHITECT Insulin assay has a sensitivity of ≤1.0μU/ml.

For peer review only - http://bmjopen.bmj.com/site/about/guidelines.xhtml

Defining MetS

MetS was defined by at least 3 of 5 metabolic syndrome diagnostic components (MetS-DCs), according to The Third Report of the National Cholesterol Education Program Expert Panel (NCEP) on Adult Treatment Panel (ATP III) Asian diagnostic criteria[23]. The five MetS-DCs were as follows: 1) SBP ≥ 130 mmHg and/or DBP ≥ 85 mmHg, or the use of anti-hypertensive drugs; 2) decreased serum HDL-C concentration < 40 mg/dl in men and <50 mg/dL in women, or treatment for dyslipidemia; 3) TG concentration ≥ 150 mg/dl, or on medication for hypertriglyceridemia; 4) hyperglycemia: fasting plasma glucose level ≥ 100 mg/dl; and 5) abdominal WC≥ 90 cm in men or ≥ 80 cm in women.

Statistical Analysis

Subjects were classified in one of three groups according to serum insulin level tertiles as the low, middle, and high insulin groups. Clinical characteristics were expressed as the mean ± standard deviation (SD) for continuous variables and number (%) for categorical variables. One-way analysis of variance (ANOVA) or a Chi-square test was used to determine p-values for continuous or categorical variables, respectively. Pearson's correlation was performed for each MetS-DC in relation to FI levels. The Cochran-Armitage trend test was used to evaluate the increasing prevalence of MetS-DCs as a function of insulin level tertile. The low FI group was designated as the reference group to calculate the ORs of the prevalence of MetS in the middle and high FI groups using multivariate logistic regression. Confounded variables present as an obstacle to valid inference in MetS studies. Hypertension and dyslipidemia are both common chronic conditions that affect a large proportion of the general adult population. Previous studies determining the association of FI and MetS also adjusted MetS-DCs[19]. Results of the adjusted model provide valid inference among MetS and insulin levels. A ROC curve was created for FI as a biomarker of MetS. The area under the ROC curve (AUC) was analyzed, and the optimized cut-off point for FI, sensitivity, and specificity were acquired

For peer review only

For peer review only - http://bmjopen.bmj.com/site/about/guidelines.xhtml

using the maximal Youden's Index. We used SPSS (version 23.0 for windows), to perform the statistical analysis. Statistical significance was set at a p-value < 0.05.

RESULTS

A total of 321 individuals, 111 men (34.6%) and 210 (65.4%) women, with a mean age of 63.91±8.32 years, were enrolled in this study. There were 90 study participants (28%) who met the diagnosis of MetS.

Table 1 shows the characteristics of the study population, which was divided based on the FI level in μU/mL. There were no statistically significant differences in age or gender between the low, middle, and high insulin level groups, while differences did exist with respect to WC, SBP, HDL-C, TG, and the proportion with MetS. Table 2 further shows the correlation between the FI level and all MetS-DCs, even after adjusting for age. FI was positively correlated with SBP, WC, FPG, and TG, and negatively correlated with HDL-C, as shown in Table 2. Table 3 shows the prevalence of MetS-DCs (hypertension, hyperglycemia, dyslipidemia, and central obesity) according to the insulin level tertiles. The prevalence of MetS-DCs increased as the FI level increased, as shown by significant p values (Cochran-Armitage trend test). Figure 1 shows that the low insulin level group had a 10% prevalence of MetS, the middle insulin level group had a 21.5% prevalence of MetS, and the high insulin level group had a 53.8% of MetS (p<0.0001 [Cochran-Armitage trend test]), suggesting that the prevalence of MetS increased with an increase in FI levels.

When designating the low insulin level group as the reference, the middle and high insulin level groups had an OR of 2.46 (p=0.02) and 10.50 (p<0.001) for MetS, respectively. After adjusting age, gender, and BMI, the middle and high insulin level groups had an OR of 1.71 (p=0.20) and 5.63 (p<0.001) for MetS, respectively. After adjusting age, gender, BMI, smoking, hypertension and dyslipidemia, the middle and high insulin level groups still had an OR of 1.51 (p=0.35) and 5.04 (p<0.001; Table 4) for MetS, respectively. There was no

For peer review only - http://bmjopen.bmj.com/site/about/guidelines.xhtml

For peer review only

significant difference between the middle and low tertile groups, but a significant difference between the high and low tertile groups, even after adjusting for the above risk factors. Based on this data, the high insulin level group had a 5-fold risk for MetS compared to the low insulin level group.

Figure 2 shows the ROC curve of FI as a biomarker for MetS. The AUC was 0.78. The optimized cut-off value for insulin was 7.35 μU/mL, with a sensitivity of 0.69 and a specificity of 0.77.

DISCUSSION

In this community-based study, we investigated fasting serum insulin levels in association with the prevalence of MetS in non-diabetic middle-aged and elderly Taiwanese adults. In our study, the prevalence of MetS in the relatively healthy middle age-to-elderly population was 28%, which is similar to the 29.75% findings reported by Li et al.[24] Among middle-aged and elderly populations in Taiwan. Looking at the three FI tertiles, there was a rising proportion of MetS as the FI level increased, also shown in previous studies[20 25 26]. This finding not only applied to MetS, but to MetS-DCs as well. The WC, SBP, TG, and FPG levels were the lowest in the low FI group and highest in the high FI group; the converse applied to HDL and vice versa (Table 1). This finding led us to speculate that an association exists between FI levels and MetS-DCs. We found a statistically significant correlation between FI levels and each MetS-DC, even after adjusting for age (Table 2). A trend existed between the FI level and the prevalence of all five MetS-DCs (Table 3). We thus wanted to know if the trend applied to the prevalence of MetS. Figure 1 shows an apparent increase in the prevalence of MetS as the FI level increased. The trend was confirmed by the Cochran-Armitage trend test ($p<0.0001$).

After adjusting for gender, age, BMI, smoking status, hypertension, and dyslipidemia, the middle-aged and elderly populations in the high FI group were at significant risk for

For peer review only - http://bmjopen.bmj.com/site/about/guidelines.xhtml

developing MetS (OR = 5.04, 95% CI =2.15-11.81; P < 0.01: Table 4). This conclusion is consistent with previous findings[20 26 27]. Not only is FI an independent risk factor for MetS, but in the cohort study of Sung et al., it was also reported that elevated FI predicted the future incidence of MetS[19]. One possible explanation might be the relationship between the FI level and insulin resistance[20 21], which has a fundamental role in MetS[28 29]. Although the mechanism by which FI may represent insulin resistance was not investigated in the present study, a number of studies have shown that FI is a suitable surrogate marker for insulin resistance[21 30-33], calculated by the fasting insulin resistance index (FIRI) or homeostasis model assessment of insulin resistance (HOMA-IR). A higher FI level is associated with insulin resistance in patients with impaired fasting glucose, but may be an inappropriate marker in diabetics with poor glycemic control. It has been reported that the FI level is highly associated with MetS[20]. In our study, the AUC for FI as an indicator for MetS was 0.78, similar to another study's AUC of 0.77[20]. Based on our search of the literature, there is no widely accepted reference range for FI. A reference range for FI of 1.57–16.32 µU/mL has been proposed in Chinese men, but the reference range varies between different ethnicities and genders[34]. A FI level above 9µU/mL has been reported to identify 80% of patients with pre-diabetes[35]. Although we obtained a cut-off value for fasting insulin, but due to large variations in insulin assays, this value of >7.35 should not be generalized to other laboratory sites.

Our findings may have an impact on health screening policies in non-diabetic people older than middle-age. Elevated FI may act as an accompanying marker to enhance the risk of MetS. We do not propose to discard MetS criteria, but suggest that elevated FI may alert physicians on the risk of MetS in clinical settings of non-diabetic individuals. Given the fact that elevated FI is not only associated with a greater risk for developing MetS[19 36] but is also associated with a greater number of cardiometabolic risk factors[22], healthy behavior should

For peer review only - http://bmjopen.bmj.com/site/about/guidelines.xhtml

be considered when the FI level is relatively higher in the population. Though we are in need of large trials to determine if subjects with early stages of insulin resistance can benefit from interventions.

Some limitations in our study merit consideration. First, the principal limitation relevant to the interpretation of our results was the use of a cross-sectional design, thus a causal relationship between the FI level and MetS cannot be inferred. Second, the sample size in our study was relatively small (n=321[the power was not calculated]) and the participants were recruited from a regional community. The participants could therefore only be distributed into three groups and the results cannot be generalized to other ethnicities. Third, though males tend to have a lower participation rate in studies[37], there may still have been a selection bias due to the higher participation of women than men in our study. Fourth, the FI cutoff value varies between different ethnic groups and insulin assays, so physicians should be aware of this variation in clinical settings. Besides, the false negative rate (31%) should be taken into consideration when applying this data. Furthermore, even though we used a standardized questionnaire, recall and reporting bias are unavoidable for self-reported data. Finally, we did not ask participants to sleep adequately or to avoid vigorous exercise the day before blood testing, which could affect the accuracy of the fasting serum insulin level.

Our study also has strengths. First, our participants were recruited during a community health examination and represent a relatively healthy population. The effects of important confounders, including ethnicity, residential area, and environmental factors, were minimized. Second, we used standardized laboratory examination protocols and anthropometric measurements. Third, while evaluating the association between MetS and the FI level, we excluded diabetic patients to avoid the effect of anti-diabetic medications on the FI level. Lastly, due to the trend of world aging, our study aimed for middle-aged and elderly populations. Studies from all around the world indicate the relationship of FI and MetS (table

For peer review only

For peer review only - http://bmjopen.bmj.com/site/about/guidelines.xhtml

5), our study contributes to the Taiwanese population.

In the future, we will continue to follow this community and record the development of newly diagnosed MetS. Counseling of healthy behaviors for residents with elevated FI will also be our topic of interest hereon. Whether lifestyle modification could retard the development of MetS in high FI individuals requires further studies to elaborate.

CONCLUSIONS

Our study provides a method to identify the risk of MetS by testing FI levels in the middle aged and elderly non-diabetic populations. When a non-diabetic individual is presented with a high FI level, physicians may be alerted of the risk of MetS. Our study confirms the association between FI and MetS. Further prospective research is needed to clarify the link between FI and MetS.

Contributors YHC and YCL were involved in writing of the manuscript. YCT, MCL, HHC and WCY conceived and supervised the study. IST provided statistical advice. JYC contributed conceived, designed and performed the experiments, collected and analyzed the data, revising it critically for important intellectual content and final approval of the version to be submitted.

Funding This work was supported by Chang Gung Memorial Hospital (CORPG3C0171, CORPG3C0172, CZRPG3C0053).

Competing interests None declared.

Ethics approval The study was approved by Chang-Gung Medical Foundation Institutional Review Board (102-2304B), and written informed consent was given by all the participants before enrollment.

Data sharing statement No additional data are available.

For peer review only

For peer review only - http://bmjopen.bmj.com/site/about/guidelines.xhtml

REFERENCES

1. Kaur J. A comprehensive review on metabolic syndrome. *Cardiol Res Pract* 2014;2014:943162. doi: 10.1155/2014/943162

2. Nakao YM, Miyawaki T, Yasuno S, et al. Intra-abdominal fat area is a predictor for new onset of individual components of metabolic syndrome: MEtabolic syndRome and abdominaL ObesiTy (MERLOT study). *Proc Jpn Acad Ser B Phys Biol Sci* 2012;88(8):454-61.

3. Popa S, Mota M, Popa A, et al. Prevalence of overweight/obesity, abdominal obesity and metabolic syndrome and atypical cardiometabolic phenotypes in the adult Romanian population: PREDATORR study. *J Endocrinol Invest* 2016;39(9):1045-53. doi: 10.1007/s40618-016-0470-4

4. Li W-C, Chen J-Y, Lin C-H, et al. Reevaluating the Diagnostic Criteria for Metabolic Syndrome in the Taiwanese Population. *Journal of the American College of Nutrition* 2011;30(4):241-47.

5. Dragsbaek K, Neergaard JS, Laursen JM, et al. Metabolic syndrome and subsequent risk of type 2 diabetes and cardiovascular disease in elderly women: Challenging the current definition. *Medicine (Baltimore)* 2016;95(36):e4806. doi: 10.1097/MD.0000000000004806

6. Meifang Y, Xue S, Jue H, et al. [Metabolic syndrome increases Framingham risk score of patients with type 2 diabetes mellitus]. *Zhejiang Da Xue Xue Bao Yi Xue Ban* 2016;45(3):268-74.

7. Oguoma VM, Nwose EU, Skinner TC, et al. Association between metabolic syndrome and 10-year risk of developing cardiovascular disease in a Nigerian population. *Int Health* 2016 doi: 10.1093/inthealth/ihw013

8. Desroches S, Lamarche B. The evolving definitions and increasing prevalence of the

For peer review only

For peer review only - http://bmjopen.bmj.com/site/about/guidelines.xhtml

metabolic syndrome. *Appl Physiol Nutr Metab* 2007;32(1):23-32. doi: 10.1139/h06-095
9. Ford ES, Giles WH, Mokdad AH. Increasing prevalence of the metabolic syndrome among u.s. Adults. *Diabetes Care* 2004;27(10):2444-9.
10. Hu G, Lindstrom J, Jousilahti P, et al. The increasing prevalence of metabolic syndrome among Finnish men and women over a decade. *J Clin Endocrinol Metab* 2008;93(3):832-6. doi: 10.1210/jc.2007-1883
11. Lim S, Shin H, Song JH, et al. Increasing prevalence of metabolic syndrome in Korea: the Korean National Health and Nutrition Examination Survey for 1998-2007. *Diabetes Care* 2011;34(6):1323-8. doi: 10.2337/dc10-2109
12. Liu M, Wang J, Jiang B, et al. Increasing Prevalence of Metabolic Syndrome in a Chinese Elderly Population: 2001-2010. *PLoS One* 2013;8(6):e66233. doi: 10.1371/journal.pone.0066233
13. Grundy SM, Brewer HB, Jr., Cleeman JI, et al. Definition of metabolic syndrome: Report of the National Heart, Lung, and Blood Institute/American Heart Association conference on scientific issues related to definition. *Circulation* 2004;109(3):433-8. doi: 10.1161/01.CIR.0000111245.75752.C6
14. Lann D, LeRoith D. Insulin resistance as the underlying cause for the metabolic syndrome. *Med Clin North Am* 2007;91(6):1063-77, viii. doi: 10.1016/j.mcna.2007.06.012
15. Gallagher EJ, Leroith D, Karnieli E. Insulin resistance in obesity as the underlying cause for the metabolic syndrome. *Mt Sinai J Med* 2010;77(5):511-23. doi: 10.1002/msj.20212
16. Dontsov AV, Vasil'eva LV. [Insulin Resistance Associated with Metabolic Syndrome as an Indicator of Cardiovascular Risk]. *Klin Med (Mosk)* 2016;94(3):189-93.
17. Polonsky KS, Given BD, Van Cauter E. Twenty-four-hour profiles and pulsatile patterns of insulin secretion in normal and obese subjects. *J Clin Invest* 1988;81(2):442-8. doi: 10.1172/JCI113339

For peer review only - http://bmjopen.bmj.com/site/about/guidelines.xhtml

18. Wilcox G. Insulin and insulin resistance. *Clin Biochem Rev* 2005;26(2):19-39.

19. Sung KC, Seo MH, Rhee EJ, et al. Elevated fasting insulin predicts the future incidence of metabolic syndrome: a 5-year follow-up study. *Cardiovasc Diabetol* 2011;10:108. doi: 10.1186/1475-2840-10-108

20. Saravia G, Civeira F, Hurtado-Roca Y, et al. Glycated Hemoglobin, Fasting Insulin and the Metabolic Syndrome in Males. Cross-Sectional Analyses of the Aragon Workers' Health Study Baseline. *PLoS One* 2015;10(8):e0132244. doi: 10.1371/journal.pone.0132244

21. ter Horst KW, Gilijamse PW, Koopman KE, et al. Insulin resistance in obesity can be reliably identified from fasting plasma insulin. *Int J Obes (Lond)* 2015;39(12):1703-9. doi: 10.1038/ijo.2015.125 [published Online First: 2015/07/15]

22. Pataky Z, Golay A, Laville M, et al. Fasting insulin at baseline influences the number of cardiometabolic risk factors and R-R interval at 3years in a healthy population: the RISC Study. *Diabetes Metab* 2013;39(4):330-6. doi: 10.1016/j.diabet.2013.05.008 [published Online First: 2013/07/24]

23. Tan CE, Ma S, Wai D, et al. Can we apply the National Cholesterol Education Program Adult Treatment Panel definition of the metabolic syndrome to Asians? *Diabetes Care* 2004;27(5):1182-6.

24. Li Z, Yang X, Yang J, et al. The Cohort Study on Prediction of Incidence of All-Cause Mortality by Metabolic Syndrome. *PLoS One* 2016;11(5):e0154990. doi: 10.1371/journal.pone.0154990

25. Kanda H, Wang P, Okamura T, et al. Fasting plasma insulin is associated with metabolic syndrome in farmers but not in nomads among the Mongolian population, China. *J Atheroscler Thromb* 2011;18(4):291-7.

26. Prevalence of the metabolic syndrome among a racially/ethnically diverse group of U.S.

For peer review only - http://bmjopen.bmj.com/site/about/guidelines.xhtml

eighth-grade adolescents and associations with fasting insulin and homeostasis model assessment of insulin resistance levels. *Diabetes Care* 2008;31(10):2020-5. doi: 10.2337/dc08-0411 [published Online First: 2008/07/02]

27. Adam FM, Nara MG, Adam JM. Fasting insulin, adiponectin, hs-CRP levels, and the components of metabolic syndrome. *Acta Med Indones* 2006;38(4):179-84.

28. Saltiel AR. Series introduction: the molecular and physiological basis of insulin resistance: emerging implications for metabolic and cardiovascular diseases. *J Clin Invest* 2000;106(2):163-4. doi: 10.1172/JCI10533

29. Mayans L. Metabolic Syndrome: Insulin Resistance and Prediabetes. *FP Essent* 2015;435:11-6.

30. Duncan MH, Singh BM, Wise PH, et al. A simple measure of insulin resistance. *Lancet* 1995;346(8967):120-1.

31. Abbasi F, Reaven GM. Evaluation of the quantitative insulin sensitivity check index as an estimate of insulin sensitivity in humans. *Metabolism* 2002;51(2):235-7.

32. Kim SH, Abbasi F, Reaven GM. Impact of degree of obesity on surrogate estimates of insulin resistance. *Diabetes Care* 2004;27(8):1998-2002.

33. Tang Q, Li X, Song P, et al. Optimal cut-off values for the homeostasis model assessment of insulin resistance (HOMA-IR) and pre-diabetes screening: Developments in research and prospects for the future. *Drug Discov Ther* 2015;9(6):380-5. doi: 10.5582/ddt.2015.01207

34. Li S, Huang S, Mo ZN, et al. Generating a reference interval for fasting serum insulin in healthy nondiabetic adult Chinese men. *Singapore Med J* 2012;53(12):821-5.

35. Johnson JL, Duick DS, Chui MA, et al. Identifying prediabetes using fasting insulin levels. *Endocr Pract* 2010;16(1):47-52. doi: 10.4158/EP09031.OR

36. Rutter MK, Sullivan LM, Fox CS, et al. Baseline levels, and changes over time in body

For peer review only

For peer review only - http://bmjopen.bmj.com/site/about/guidelines.xhtml

mass index and fasting insulin, and their relationship to change in metabolic trait clustering. *Metab Syndr Relat Disord* 2014;12(7):372-80. doi: 10.1089/met.2013.0148

37. Galea S, Tracy M. Participation rates in epidemiologic studies. *Ann Epidemiol* 2007;17(9):643-53. doi: 10.1016/j.annepidem.2007.03.013 [published Online First: 2007/06/08]

Figure Legends

Figure 1. Prevalence of metabolic syndrome based on insulin levels. A linear increasing trend across insulin tertiles.

Figure 2. ROC curve for insulin as a biomarker of metabolic syndrome. AUC 0.78. FI 7.35μU/mL (sensitivity: 0.69; specificity: 0.77).

For peer review only

For peer review only - http://bmjopen.bmj.com/site/about/guidelines.xhtml

Table 1 General characteristics of the study population based on insulin levels.

	Insulin levels				
	**Total**	**Low**	**Middle**	**High**	
**Variables**	**n=321**	**n=110 (≦4.8)**	**n=107 (4.9-7.8)**	**n=104 (≧7.9)**	**p value**
Age (year)	63.91 ±8.32	64.23 ±8.32	64.47 ±8.67	63.01 ±7.93	0.40
BMI (kg/m^2)	24.36 ±3.53	22.41 ±3.14	24.41 ±2.73	26.37 ±3.54	<0.001
Waist circumference (cm)	84.23 ±9.51	79.69 ±7.57	83.78 ±8.63	89.51 ±9.65	<0.001
SBP (mmHg)	129.02 ±16.59	123.69 ±17.36	129.52 ±14.51	134.13 ±16.20	<0.001
DBP (mmHg)	77.01 ±10.90	75.43 ±11.80	76.92 ±10.03	78.79 ±10.60	0.08
ALT (U/L)	21.74 ±11.06	18.94 ±7.81	20.33 ±9.25	26.15 ±14.05	<0.001
Creatinine (mg/dL)	0.76 ±0.44	0.69 ±0.17	0.85 ±0.66	0.75 ±0.34	0.03
FPG (mg/dL)	89.10 ±9.93	85.29 ±9.11	89.18 ±8.52	93.05 ±10.60	<0.001
HDL-C (mg/dL)	55.70 ±14.05	60.93 ±14.85	55.59 ±13.17	50.28 ±11.94	<0.001
Insulin (μU/mL)	7.10 ±4.14	3.60 ±0.94	6.21 ±0.86	11.72 ±4.02	<0.001
LDL-C (mg/dL)	121.48 ±32.05	118.90 ±34.55	126.03 ±31.01	119.53 ±30.10	0.20
T-cholesterol (mg/dL)	200.61 ±35.20	198.85 ±36.98	203.81 ±35.12	119.18 ±33.43	0.52
TG (mg/dL)	117.34 ±60.61	95.39 ±45.13	111.04 ±49.83	147.05 ±72.48	<0.001
Current smoking, n(%)	34 (10.6)	14 (12.7)	11 (10.3)	9 (8.7)	0.62
Marital status (single), n(%)	54 (16.8)	22 (20.0)	14 (13.1)	18 (17.3)	0.39
Men, n(%)	111 (34.6)	41 (37.3)	39 (36.4)	31 (29.8)	0.46
Regular exercise, n(%)	264 (82.2)	92 (83.6)	96 (89.7)	76 (73.1)	0.01
Vegetarian, n(%)	20 (6.2)	7 (6.4)	7 (6.5)	6 (5.8)	0.97
HTN, n(%)	150 (46.7)	43 (39.1)	47 (43.9)	60 (57.7)	0.02
Hyperlipidemia, n(%)	204 (63.6)	58 (52.7)	69 (64.5)	77 (74.0)	0.005
Metabolic syndrome, n(%)	90 (28.0)	11 (10.0)	23 (21.5)	56 (53.8)	<0.001

Notes: Ranges of FI levels of different tertile groups are shown in brackets at the top of the table, units in μU/mL.

Clinical characteristics are expressed as the mean±SD for continuous variables and n(%) for categorical variables. P-values were derived from one-way analysis of variance (ANOVA) for

For peer review only

395 continuous variables and chi-square test for categorical variables.

396 **Abbreviations:** BMI, body mass index; SBP, systolic blood pressure; DBP, diastolic blood
397 pressure; ALT, alanine aminotransferase; FPG, fasting plasma glucose; HDL-C, high-density
398 lipoprotein cholesterol; LDL-C, low-density lipoprotein cholesterol; TG, triglyceride; HTN,
399 hypertension.

400

401

402

403

404 **Table 2** Pearson's correlation coefficients for each component of metabolic syndrome and age
405 in relation to insulin levels.

Variables	Insulin(n=321)			
	Unadjusted		Adjusted for age	
	Pearson's coefficient	p value	Pearson's coefficient	p value
Age (year)	-0.04	0.50	NA	NA
SBP (mmHg)	0.21	<0.001	0.22	<0.001
DBP (mmHg)	0.11	0.05	0.10	0.07
Waist circumference (cm)	0.43	<0.001	0.44	<0.001
FPG (mg/dL)	0.38	<0.001	0.39	<0.001
HDL-C (mg/dL)	-0.37	<0.001	-0.37	<0.001
TG (mg/dL)	0.37	<0.001	0.37	<0.001

406 **Abbreviations:** SBP, systolic blood pressure; DBP, diastolic blood pressure; FPG, fasting
407 plasma glucose; HDL-C, high-density lipoprotein cholesterol; TG, triglyceride.

For peer review only - http://bmjopen.bmj.com/site/about/guidelines.xhtml

Table 3 Prevalence of components of metabolic syndrome based on insulin levels.

**Components**	**Low (n=110) N(%)**	**Middle (n=107) N(%)**	**High (n=104) N(%)**	**p value for Cochran-Armitage trend test**
High blood pressure[a]	56(50.9)	63(58.9)	78(75)	0.0003
High blood glucose[b]	8(7.3)	10(9.3)	25(24.0)	0.0004
Low HDL-C[c]	15(13.6)	19(17.8)	43(41.3)	<0.0001
High TG[d]	17(15.5)	27(25.2)	42(40.4)	<0.0001
Central obesity[e]	34(30.9)	57(53.3)	82(78.8)	<0.0001

Note:

[a] SBP≧130 mmHg or DBP≧85 mmHg, or self-reported hypertension

[b] Fasting blood glucose ≥100 mg/dL or self-reported diabetes mellitus

[c] HDL-C<40 mg/dL in men or <50 mg/dL in women

[d] TG≥150 mg/dL

[e] Waist circumference ≥90 cm in men or ≥80 cm in women

Abbreviations: HDL-C, high-density lipoprotein-cholesterol; TG, triglyceride; SBP, systolic blood pressure; DBP, diastolic blood pressure.

For peer review only

For peer review only - http://bmjopen.bmj.com/site/about/guidelines.xhtml

Table 4 Association between insulin levels and metabolic syndrome.

Variables	Model 1			Model 2			Model 3		
	OR	(95%CI)	p value	OR	(95%CI)	p value	OR	(95%CI)	p value
Low	1.00	—	—	1.00	—	—	1.00	—	—
Middle	2.46	(1.14-5.35)	0.02	1.71	(0.76-3.85)	0.20	1.51	(0.64-3.57)	0.35
High	10.50	(5.05-21.84)	<0.001	5.63	(2.53-12.53)	<0.001	5.04	(2.15-11.81)	<0.001
p value for trend			<0.001			<0.001			<0.001

Model 1: unadjusted

Model 2: adjusted for gender, age, and BMI

Model 3: adjusted for factors in model 2 plus smoking, HTN, and hyperlipidemia

Abbreviations: BMI, body mass index; HTN, hypertension; OR, odds ratio; CI, confidence interval.

For peer review only

For peer review only - http://bmjopen.bmj.com/site/about/guidelines.xhtml

Table 5 Studies of Association Between Fasting Insulin and Metabolic Syndrome.

Authors	Study Year	Study Population	% MetS	Fasting insulin	Risk of MetS	Main Finding	Reference
Saravia G et al 2015	2009-2010 Cross sectional	3200 Non-diabetic males in Spain	23%	Highest tertile (≥6.13) vs lowest (≤3.80)μU/mL	OR (95%CI) 11.36 (8.65-15.13) for MetS	Per each 10 pmol/L (1.4 uU/mL) increase in insulin, the odds for metabolic syndrome increased by 1.43 (95%CI: 1.38, 1.49)	[20]
Rutter MK et al 2014	(1991-1995) to (1998-2001) 7 year Prospective	2616 non-diabetic adults in Europe	-	1-quintile change in fasting insulin (pmol/L)	mean (95% CI) 0.12 (0.10–0.15) [MetS trait score 7 year change]	Change in metabolic trait clustering was significantly associated with baseline levels and changes in fasting insulin.	[38]
Sung KC et al 2011	2003-2008 5 year cohort	2350 non-MetS in Korea	8.5% (incidence)	Highest quartile (≥8.98) vs lowest (≤6.01) IU/ml	OR (95% CI) of developing MS 5.1 (3.1-8.2)	The highest quartile of the insulin levels had more than a 5 times greater risk of developing MS compared to the subjects in the lowest quartile.	[19]
Kanda H et al 2011	2000,2001 Cross sectional	456 in Mongolia	6.4%	Highest tertile (≥10.33) vs lowest (≤6.72) mmol/L	Percentage of MetS 17.1% vs 4.6%	Fasting plasma insulin is associated with MetS in farmers, but not nomads among the Mongolian population in China.	[27]
STOPP-T2D PSG* 2008	2003 Cross sectional	1453 8th grade adolescents in the U.S.	9.5%	Highest quintile (≥39.1) vs lowest (≤17.0) μU/mL	OR (95%CI) 199.64(31.29-1273.7) for MetS	The highest insulin quintile were almost 200 times more likely to be classified with the metabolic syndrome than participants in the lowest quintile.	[28]
Adam FM et al 2006	2005 Cross sectional	128 overweight/ obese in Indonesia	68.8%	Mean fasting insulin levels15.68±7.85 vs 3.16±2.53 (uU/ml) with 5 components vs 1 component of MetS.		There is a strong linear increase in fasting insulin levels with an increase of the number of metabolic syndrome.	[29]

*STOPP-T2D PSG: Studies to Treat or Prevent Pediatric Type 2 Diabetes Prevention Study Group

For peer review only - http://bmjopen.bmj.com/site/about/guidelines.xhtml

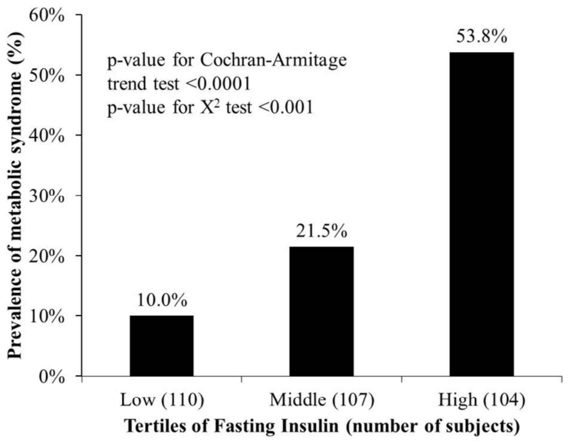

Figure 1 Prevalence of metabolic syndrome based on insulin levels. A linear increasing trend across insulin tertiles.

Figure 1. Prevalence of metabolic syndrome based on insulin levels. A linear increasing trend across insulin tertiles.

297x209mm (300 x 300 DPI)

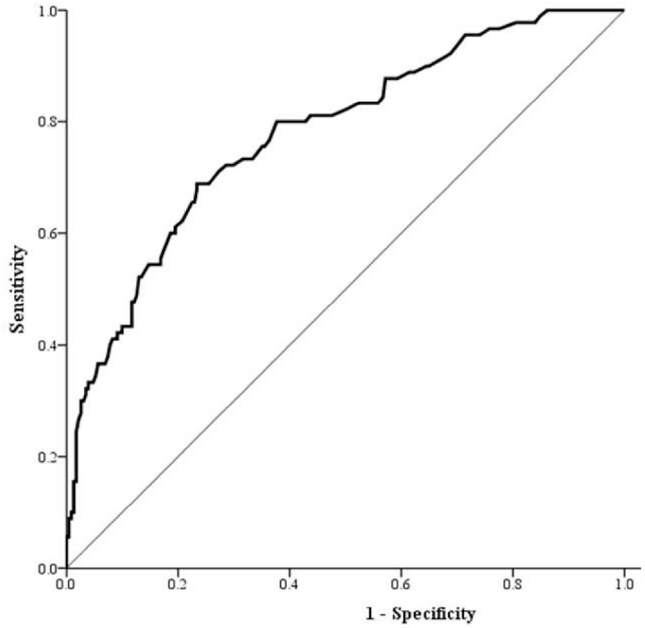

Figure 2. ROC curve for insulin as a biomarker of metabolic syndrome. AUC 0.78. FI 7.35µU/mL (sensitivity: 0.69; specificity: 0.77).

Figure 2. ROC curve for insulin as a biomarker of metabolic syndrome. AUC 0.78. FI 7.35µU/mL (sensitivity: 0.69; specificity: 0.77).

297x209mm (300 x 300 DPI)

For peer review only - http://bmjopen.bmj.com/site/about/guidelines.xhtml

STROBE 2007 (v4) Statement—Checklist of items that should be included in reports of *cross-sectional studies*

Section/Topic	Item #	Recommendation	Reported on page #
**Title and abstract**	1	(*a*) Indicate the study's design with a commonly used term in the title or the abstract	P2
		(*b*) Provide in the abstract an informative and balanced summary of what was done and what was found	P2
**Introduction**			
Background/rationale	2	Explain the scientific background and rationale for the investigation being reported	P4
Objectives	3	State specific objectives, including any prespecified hypotheses	P2, P4
**Methods**			
Study design	4	Present key elements of study design early in the paper	P4
Setting	5	Describe the setting, locations, and relevant dates, including periods of recruitment, exposure, follow-up, and data collection	P4, 5
Participants	6	(*a*) Give the eligibility criteria, and the sources and methods of selection of participants	P4
Variables	7	Clearly define all outcomes, exposures, predictors, potential confounders, and effect modifiers. Give diagnostic criteria, if applicable	P5
Data sources/ measurement	8*	For each variable of interest, give sources of data and details of methods of assessment (measurement). Describe comparability of assessment methods if there is more than one group	P5
Bias	9	Describe any efforts to address potential sources of bias	Nil
Study size	10	Explain how the study size was arrived at	Nil
Quantitative variables	11	Explain how quantitative variables were handled in the analyses. If applicable, describe which groupings were chosen and why	
Statistical methods	12	(*a*) Describe all statistical methods, including those used to control for confounding	P6
		(*b*) Describe any methods used to examine subgroups and interactions	P6
		(*c*) Explain how missing data were addressed	Nil
		(*d*) If applicable, describe analytical methods taking account of sampling strategy	Nil
		(*e*) Describe any sensitivity analyses	Nil
**Results**			

For peer review only

Participants	13*	(a) Report numbers of individuals at each stage of study—eg numbers potentially eligible, examined for eligibility, confirmed eligible, included in the study, completing follow-up, and analysed	Nil
		(b) Give reasons for non-participation at each stage	Nil
		(c) Consider use of a flow diagram	Nil
Descriptive data	14*	(a) Give characteristics of study participants (eg demographic, clinical, social) and information on exposures and potential confounders	P8
		(b) Indicate number of participants with missing data for each variable of interest	Nil
Outcome data	15*	Report numbers of outcome events or summary measures	P6-9
Main results	16	(*a*) Give unadjusted estimates and, if applicable, confounder-adjusted estimates and their precision (eg, 95% confidence interval). Make clear which confounders were adjusted for and why they were included	P7
		(*b*) Report category boundaries when continuous variables were categorized	P8
		(*c*) If relevant, consider translating estimates of relative risk into absolute risk for a meaningful time period	Nil
Other analyses	17	Report other analyses done—eg analyses of subgroups and interactions, and sensitivity analyses	Nil
**Discussion**			
Key results	18	Summarise key results with reference to study objectives	P7
Limitations	19	Discuss limitations of the study, taking into account sources of potential bias or imprecision. Discuss both direction and magnitude of any potential bias	P17
Interpretation	20	Give a cautious overall interpretation of results considering objectives, limitations, multiplicity of analyses, results from similar studies, and other relevant evidence	P17
Generalisability	21	Discuss the generalisability (external validity) of the study results	P18
**Other information**			
Funding	22	Give the source of funding and the role of the funders for the present study and, if applicable, for the original study on which the present article is based	P19

*Give information separately for cases and controls in case-control studies and, if applicable, for exposed and unexposed groups in cohort and cross-sectional studies.

Note: An Explanation and Elaboration article discusses each checklist item and gives methodological background and published examples of transparent reporting. The STROBE checklist is best used in conjunction with this article (freely available on the Web sites of PLoS Medicine at http://www.plosmedicine.org/, Annals of Internal Medicine at http://www.annals.org/, and Epidemiology at http://www.epidem.com/). Information on the STROBE Initiative is available at www.strobe-statement.org.

For peer review only
